# New Antibacterial Bagremycins F and G from the Marine-Derived *Streptomyces* sp. ZZ745

**DOI:** 10.3390/md16090330

**Published:** 2018-09-12

**Authors:** Di Zhang, Chenyan Shu, Xiaoyuan Lian, Zhizhen Zhang

**Affiliations:** 1 Ocean College, Zhoushan Campus, Zhejiang University, Zhoushan 316021, China; zhangdi163.love@163.com; 2 College of Pharmaceutical Sciences, Zhejiang University, Hangzhou 310058, China; 21519023@zju.edu.cn

**Keywords:** *Streptomyces* sp. ZZ745, bagremycin F, bagremycin G, antibacterial activity

## Abstract

As part of our research to discover novel bioactive natural products from marine microorganisms, five bagremycin analogues, including the previously unreported bagremycins F (**1**) and G (**2**), were isolated from a marine actinomycete *Streptomyces* sp. ZZ745. The structures of these compounds were determined by means of NMR spectroscopic analysis, HRESIMS data, and optical rotation. Both bagremycins F (**1**) and G (**2**) showed antibacterial activity against *Escherichia coli*, with MIC values of 41.8 and 61.7 μM, respectively.

## 1. Introduction

More than 60% of new drugs with small molecules are derived from natural products, indicating that natural products are important sources for the discovery and development of new drugs [1]. Marine actinomycetes are one of the richest producers of bioactive natural products [2,3,4,5]. For example, salinosporamide A from the marine *Salinospora* strain CNB-392 entered clinical trials against multiple myeloma only three years after its discovery [6], and abyssomicin C from marine *Verrucosispora* strain AB 18-032 is the first natural product that inhibits *p*-aminobenzoate biosynthesis, a pathway used only by microorganisms [7].

Bagremycins are bacterial secondary metabolites and belong to phenol esters formed from *p*-hydroxystyrene and *p*-hydroxybenzoic acid. The first two bagremycins A and B, from the soil actinomycete *Streptomyces* sp. Tü 4128, were reported in 2001 [8]. Since then, no new bagremycins have been reported until our recent publication, which reported three new bagremycins C–E from a mangrove-derived actinomycete *Streptomyces* sp. Q22 [9]. Both bagremycins A and B showed antibacterial activity against *Arthrobacter aurescens* DSM20166 and *Streptomyces viridochromogenes* Tü 57 [8]. Bagremycin C inhibited the proliferation of different glioma cell lines; induced apoptosis in human glioma U87-MG cells in a dose- and time-dependent manner; and arrested the U87-MG cell cycle at the G_0_/G_1_ phase [9]. The biosynthesis of bagremycins was also investigated, and several genes including bagA, bagB, bagC, and bagI are involved in bagremycin biosynthesis [10,11,12].

**Figure 1:**
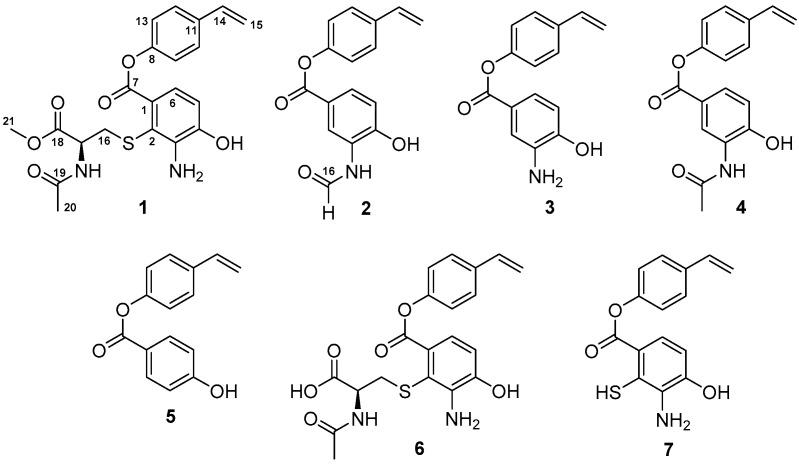
Structures of **1**–**7**.

As part of our research for discovering novel bioactive compounds from marine-derived microorganisms [9], a marine strain ZZ745 was isolated from a coastal mud. A scale up culture of the strain ZZ745 in Gause’s liquid medium produced two new members of bagremycins, named bagremycins F (**1**) and G (**2**), together with the known bagremycins A (**3**), B (**4**), and E (**5**) (Figure 1). Herein, we reported the isolation and culture of the strain ZZ745, as well as the isolation, structural elucidation, and antibacterial activity of the isolated compounds.

## 2. Results and Discussion

The strain ZZ745 (Supplementary materials, Figure S1) isolated from a sample of coastal mud was identified as *Streptomyces* sp. ZZ745, according to the results from its 16S rDNA sequence analysis (Supplementary materials, Table S1 and Figure S2). A crude extract prepared from a 50 L culture of the strain ZZ745 in Gause′s liquid medium was fractionated by repeated column chromatography, followed by purification with high performance liquid chromatography (HPLC) to give **1**–**5**. On the basis of the NMR spectral analysis and comparison of their NMR data with those reported from the literatures, **3**–**5** were identified as bagremycins A (**3**), B (**4**), and E (**5**) [8,9]. Their ^13^C and ^1^H NMR data are shown in the Supplementary materials (Supplementary materials, Table S2).

**Figure 2:**
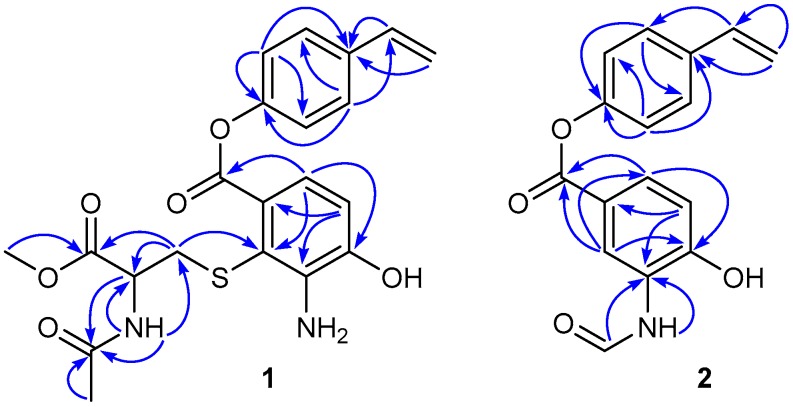
Key HMBC correlations of bagremycins F (**1**) and G (**2**).

Compound **1** was obtained as a colorless amorphous powder and gave ion peaks at *m*/*z* 431.1270 [M + H]^+^ and 453.1089 [M + Na]^+^ in the (+)-HRESIMS, corresponding to a molecular formula of C_21_H_22_N_2_O_6_S. The ^13^C NMR spectrum (Supplementary materials, Figure S4) of **1** showed 21 carbon signals, fifteen of which were attributed to the scaffold of **1**, resonating at *δ*_C_ 126.5 (C-1), 113.1 (C-2), 140.4 (C-3), 147.7 (C-4), 113.2 (C-5), 119.1 (C-6), 165.7 (C-7), 150.5 (C-8), 122.0 (C-9, C-13), 127.1 (C-10, C-12), 134.7 (C-11), 135.8 (C-14), and 114.3 (C-15). The ^1^H NMR spectrum (Supplementary materials, Figure S3) displayed the signal at *δ*_H_ 6.78 (1H, d, *J* = 8.2 Hz, H-5); 7.10 (1H, d, *J* = 8.2 Hz, H-6); 7.21 (2H, d, *J* = 8.6 Hz, H-9, H-13); 7.55 (2H, d, *J* = 8.6 Hz, H-10, H-12); 6.74 (1H, dd, *J* = 17.7, 10.9 Hz, H-14); 5.29 (1H, dd, *J* = 10.9, 0.6 Hz, Ha-15); and 5.82 (1H, dd, *J* = 17.7, 0.6 Hz, Hb-15) (Table 1). The ^1^H and ^13^C NMR data for the scaffold of **1** were very similar to those of bagremycins C (**6**) and D (**7**) [9], suggesting that the core structures of **1**, **6**, and **7** had the same substitution pattern. The remaining six carbons were assigned to an *N*-acetylcysteine methyl ester moiety, comprising of the following ^13^C and ^1^H NMR signals: *δ*_C_ 35.5 (*δ*_H_ 2.98, dd, *J* = 13.1, 8.4 Hz and 3.11, dd, *J* = 13.1, 5.4 Hz, CH_2_-16); *δ*_C_ 52.2 (*δ*_H_ 4.25, m, NH-CH-17); *δ*_C_ 171.0 and 169.3 (C-18 and C-19); *δ*_C_ 22.2 (*δ*_H_ 1.81, s, CH_3_-20); *δ*_C_ 51.9 (*δ*_H_ 3.54, s, CH_3_-21) and (*δ*_H_ 8.41, d, *J* = 7.5 Hz, NH). The significant HMBC correlations (Figure 2) from H_2_-16 to C-2 (*δ* 113.1), placed the *N*-acetylcysteine methyl ester on C-2. Since both **1** and **6** are not only levorotatory, but also have nearly identical structures, it was legitimate to assume that both compounds had the same absolute configuration at C-17 of the amino acid moiety. Since the absolute configuration of C-17 of **6** was determined as 17*S* [9], it was concluded that the configuration of C-17 of **1** was also 17*S*. The structure of **1** was a new member of bagremycins, named bagremycin F. Its ^13^C and ^1^H NMR data were fully assigned by the HSQC and HMBC correlations (Table 1 and Figure 2).

Since bagremycin F (**1**) was a methyl ester of bagremycin C (**6**), it was possible that **1** could be an artifact formed by esterification of the carboxylic acid of **6** by MeOH, which was used as the solvent for extraction. To prove this possibility, an ethanol extract prepared from the mycelia of the strain ZZ745 was analyzed by HPLC-HRESIMS. The results (Supplementary materials, Figures S23 and S24) showed that bagremycin F (**1**) was detected in the ethanol crude extract. Therefore, it was clear that **1** was not an artifact, but a new naturally occurring compound.

The molecular formula of C_16_H_13_NO_4_ for **2** was deduced from the (+)-HRESIMS at *m*/*z* 284.0929 [M + H]^+^ and 306.0734 [M + Na]^+^, as well as its ^13^C data. The ^13^C and ^1^H NMR spectra (Supplementary materials, Figures S10 and S11) of **2** showed the signals for one *p*-hydroxybenzoic acid ester carbonyl (*δ*_C_ 164.3), one formamide unit (*δ*_C_ 160.2; *δ*_H_ 8.33, 9.78), twelve aromatic carbons and seven aromatic protons, including: *δ*_C_ 118.1 (C-1), 121.9 (C-2), 126.2 (C-3), 152.6 (C-4), 114.8 (C-5), 126.9 (C-6), 150.4 (C-8), 122.1 (C-9, C-13), 127.1 (C-10, C-12), 134.7 (C-11), and *δ*_H_ 8.90 (d, *J* = 2.2 Hz, H-2); 6.99 (d, *J* = 8.5 Hz, H-5); 7.74 (dd, *J* = 8.5, 2.2 Hz, H-6); 7.20 (d, *J* = 8.6 Hz, H-9, H-13); 7.54 (d, *J* = 8.6 Hz, H-10, H-12); and an exocyclic double bond (*δ*_C_ 135.8, 114.3; *δ*_H_ 6.74, 1H, dd, *J* = 17.6, 10.9 Hz, 5.82, 1H, dd, *J* = 17.6, 0.7 Hz, 5.27, 1H, dd, *J* = 10.9, 0.7 Hz) (Table 1). These NMR data indicated that **2** was an analogue of bagremycins. The molecular formula, together with the ^1^H and ^13^C NMR data of **2**, revealed that its structure was nearly identical to that of **4,** except for the acetyl group in **4**, which was replaced by the formyl group in **2**. Literature search revealed that **2** was a new member of bagremycins, which was named bagremycin G.

**Figure 3:**
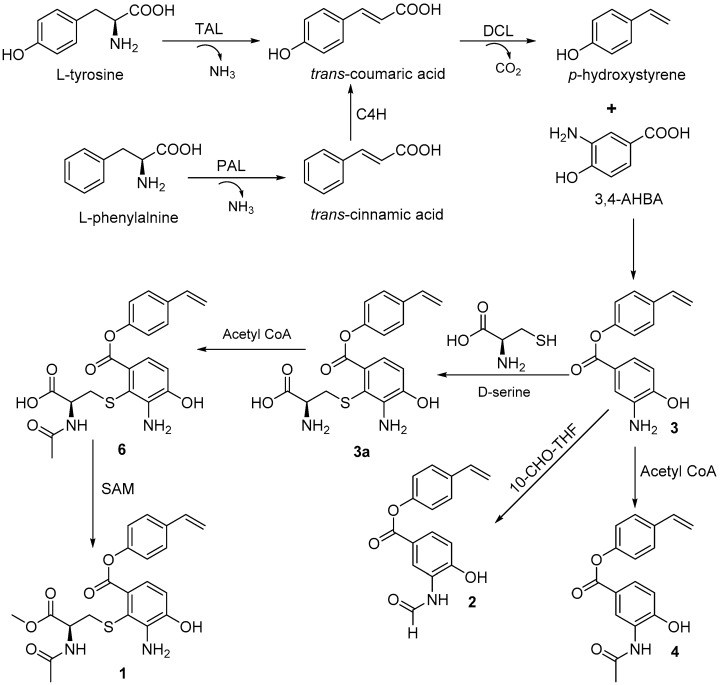
Proposed biosynthetic pathway of bagremycins (C4H: cinnamate 4-hydroxylase; 10-CHO-THF: 10-formyltetrahydrofolate [13,14]; DCL: decarboxylase; PAL: phenylalanine ammonia lyase; SAM: S-adenosylmethionine; TAL: tyrosine ammonia lyase; 3,4-AHBA: 3-amino-4-hydroxybenzoic acid).

The biosynthetic pathway for bagremycins A (**3**) and B (**4**) was previously proposed, as described in Reference [10]. They are the condensation products of 3-amino-4-hydroxybenzoic acid (3,4-AHBA) and *trans*-coumaric acid, which is derived from l-tyrosine or l-phenylalanine. Accordingly, bagremycins F (**1**) and G (**2**) should share the same biosynthetic pathway as that of bagremycins A (**3**) and B (**4**) and may be derived from bagremycin A (**3**) (Figure 3).

The antibacterial activity of **1**–**5** against methicillin-resistant *Staphylococcus aureus* (MRSA), *Escherichia coli*, and *Candida albicans* was evaluated using the micro broth dilution method [15]. Gentamicin (an antibiotic against both Gram-positive and Gram-negative bacteria) and amphotericin B (an antifungal drug) were used as positive controls. As shown in Table 2, bagremycins F (**1**) and G (**2**) showed antibacterial activity against *E. coli*, with MIC values of 41.8 and 67.1 μM, respectively, but were inactive against MRSA and *C. albicans* in a concentration of 116.2 μM for **1** and 176.5 μM for **2**.

Bagremycin C (**6**) was previously reported to have strong growth inhibitory activity against glioma cells [9]. Therefore, bagremycins F (**1**) and G (**2**) were also evaluated for their activity against human glioma U87-MG and U251 cell lines by sulforhodamine B (SRB) assay [9,16]. Doxorubicin (DOX) was used as a positive control. The results showed that both bagremycins F (**1**) and G (**2**) were not active against both cell lines, even at a concentration as high as 100 μM.

## 3. Materials and Methods

### 3.1. General Experimental Procedures

Ultraviolet-visible (UV) and infrared radiation (IR) spectra were recorded on a METASH UV-8000 spectrometer (Shanghai METASH Instruments Co. Ltd., Shanghai, China) and a Bruker TENSOR II high performance FT-IR spectrometer (Bruker, Karlsruhe, Germany), respectively. Optical rotation was measured on an Autopol I polarimeter (Rudolph Research Analytical, Hackettstown, NJ, USA). HRESIMS data was obtained from an Agilent 6230 time-of-flight liquid chromatography–mass spectrometry (TOF LC-MS) (Agilent, CA, USA). NMR spectra were acquired on a Bruker 500 spectrometer (Fällanden, Switzerland), and chemical shifts were expressed in *δ* (ppm). Sephadex LH-20 (GE Healthcare, Stockholm, Sweden) and octadecyl-functionalized silica gel (ODS, Cosmosil 75C_18_-Prep, Nacalai Tesque Inc., Kyoto, Japan) were used for column chromatography. HPLC separation was conducted on an Agilent 1260 HPLC system with a diode array detector (DAD), using a Zorbax SB-C_18_ column (250 × 9.4 mm, 5 μm, Agilent Technologies, Palo Alto, CA, USA). All solvents were purchased from the Shanghai Lingfeng Co., Ltd. (Shanghai, China). Amphotericin B (>95.0%) and gentamicin (99.6%) were obtained from Meilune Biotechnology Co. Ltd. (Dalian, China), and doxorubicin (DOX, >98.0%) from Sigma-Aldrich. Human glioma U87-MG and U251 cells were obtained from the Cell Bank of the Chinese Academy of Sciences. Gause’s medium was bought from Guangdong Huankai Microbial Science and Technology Co. Ltd. (Guangzhou, China). Methicillin-resistant *Staphylococcus aureus* ATCC 43300 (MRSA), *Escherichia coli* ATCC 25922, and *Candida albicans* were gifts from Professors Zhongjun Ma, Pinmei Wang, and Bin Wu, respectively.

### 3.2. Isolation and Identification of Strain ZZ745

Strain ZZ745 was isolated from a sample of marine mud, which was collected from a coastal area located at the Jintang Island of Zhoushan (Zhejiang, China), in September 2016. Summarily, soil (1.0 g) was transferred into a sterile tube containing 9 mL sterile sea water to make a 1 × 10^−1^ g/mL suspension, after shaking for 10 min on a rotary shaker at 180 rpm. A diluted suspension (1 × 10^−3^ g/mL, 200 μL) from the 1 × 10^−1^ g/mL suspension was dispersed evenly on the surface of Gause’s agar medium in a plate, and then incubated for seven days at room temperature. The single colony (strain ZZ745) was shifted onto the surface of Gause’s agar medium in another plate, and then incubated for another six days for securing pure strain. The pure strain ZZ745 was finally transferred onto slant with Gause’s agar medium, and then stored at 4 °C for further use.

The strain ZZ745 was identified by 16S rDNA sequence analysis, which was conducted by Legenomics (Hangzhou, China). Its 16S rDNA sequence has been deposited in GenBank (accession number: MH734530). A voucher strain (*Streptomyces* sp. ZZ745) was preserved at the Laboratory of the Institute of Marine Biology, Ocean College, Zhejiang University, China.

### 3.3. Large Scale Culture of Strain ZZ745

The pure colony of strain ZZ745 from slant was refreshed and inoculated into 250 mL Gause’s liquid medium in a 500 mL Erlenmeyer flask, which was cultured at 28 °C for five days in a shaker at 180 rpm, to make seed broth. The seed broth of 5 mL was transferred into a 500 mL Erlenmeyer flask containing 250 mL Gause’s liquid medium, and then cultured for 21 days at the same conditions for the culture of seed broth. A total of 50 L culture was prepared for this study.

### 3.4. Isolation of Compounds ***1***–***5***

The total 50 L culture was separated into two parts of mycelia and supernatant by centrifugation. The mycelia were extracted with MeOH (3 × 300 mL). The methanolic solutions were combined and evaporated under reduced pressure to give a crude extract A (3.3 g). The supernatant was partitioned with EtOAc (3 × 25 L), and the EtOAc solutions were combined and evaporated under reduced pressure to afford a crude extract B (5.5 g). A combination of the extracts A and B (8.8 g) was fractionated by an ODS column eluting with 20%, 40%, 60%, 80%, and 100% MeOH, to give five fractions A–E. The fraction C was further separated by a Sephadex LH-20 column eluting with 50% MeOH to furnish subfractions C_1_ and C_2_, based on the results from thin layer chromatography (TLC) analysis. Compounds **1** (1.2 mg, t_R_ 30.2 min, acetonitrile:H_2_O, 48:52) and **2** (1.3 mg, t_R_ 34.4 min, MeOH:H_2_O, 60:40) were obtained from the subfractions C_1_ and C_2_, respectively, by HPLC purification through a Zorbax SB-C_18_ column (250 × 9.4 mm, 5 μm). Similarly, the fraction D was separated by an ODS column eluting with 75% MeOH to give subfractions D_1_ and D_2_, based on the results from TLC analysis. By HPLC purification using the same Zorbax SB-C_18_ column, **3** (8.2 mg, t_R_ 18.5 min, MeOH:H_2_O, 75:25) and **4** (22.1 mg, t_R_ 24.3 min, MeOH:H_2_O, 75: 25) were purified from the subfraction D_1_, and **5** (19.8 mg, t_R_ 29.2 min, MeOH:H_2_O, 80:20) from the subfraction D_2_.

**Bagremycin F (1)**: colorless amorphous powder; molecular formula C_21_H_22_N_2_O_6_S; [α]${}_{D}^{20}$ −14.0 (*c* 0.04, MeOH); UV (MeOH) λ_max_ (log ε) 201 (3.49), 250 (2.92), 331 (2.34) nm; IR (KBr) *ν*_max_ 3317, 2921, 2853, 1714, 1461, 1254, 1171, 1033 cm^−1^; ^13^C and ^1^H NMR data (in DMSO-*d*_6_), see Table 1, HRESIMS *m*/*z* 431.1270 [M + H]^+^ (calcd. for C_21_H_23_N_2_O_6_S, 431.1277) and 453.1089 [M + Na]^+^ (calcd. for C_21_H_22_N_2_NaO_6_S, 453.1096).

**Bagremycin G (2)**: colorless amorphous powder; molecular formula C_16_H_13_NO_4_; UV (MeOH) λ_max_ (log ε) 201 (3.14), 247 (2.97), 321 (1.92) nm; IR (KBr) *ν*_max_ 3380, 2918, 2853, 1714, 1540, 1464, 1256, 1198, 1050 cm^−1^; ^13^C and ^1^H NMR data (in DMSO-*d*_6_), see Table 1, HRESIMS *m*/*z* 284.0929 [M + H]^+^ (calcd. for C_16_H_14_NO_4_, 284.0923) and 306.0734 [M + Na]^+^ (calcd. for C_16_H_13_NNaO_4_, 306.0742).

### 3.5. Antimicrobial Activity Assay

The antimicrobial activity of **1**–**5** against MRSA, *E. coli*, and *C. albicans* was determined by the micro broth dilution method, as described in the previous study in Reference [15].

### 3.6. Antitumor Activity Assay

The activity of **1**–**5** against human glioma U87-MG and U251 cells was evaluated by SRB assay and DOX was used as a positive control. U87-MG and U251 cells were cultured in MEM (Minimum Essential Medium, Gibco, Grand Island, NY, USA) and DMEM (Dulbecco’s Modified Eagle Medium, Gibco, Grand Island, NY, USA), both adding 10% FBS (Fetal Bovine Serum, PAA Laboratories Inc., Toronto, ON, Canada), respectively. All glioma cells were incubated at 37 °C in a humidified incubator with 5% CO_2_. Cells after the third generation were used for the experiment. The detailed procedure for SRB assay was described in previous publications in References [9,16].

## 4. Conclusions

Marine actinomycetes, especially from the *Streptomyces* genus, are important sources for the discovery of novel bioactive natural products. In this study, we isolated and identified five bagremycin derivatives (**1**–**5**) from the culture of the marine-derived actinomycete *Streptomyces* sp. ZZ745 in Gause’s liquid medium. Bagremycins F (**1**) and G (**2**) are the two new members of bagremycins, which enriched the structural diversity of the bagremycin family. Bagremycins F (**1**) and G (**2**) showed antibacterial activity against *E. coli*.

## Figures and Tables

**Table 1:** ^13^C (125 MHz) and ^1^H (500 MHz) NMR data of bagremycins F (**1**) and G (**2**) (in DMSO-*d*_6_).

No.	1			2		
	*δ*_C_, Type	*δ*_H_ (*J* in Hz)	HMBC	*δ*_C_, Type	*δ*_H_ (*J* in Hz)	HMBC
1	126.5, C			118.1, C		
2	113.1, C			121.9, CH	8.90, d (2.2)	C_3_, C_4_, C_6_, C_7_
3	140.4, C			126.2, C		
4	147.7, C			152.6, C		
5	113.2, CH	6.78, d (8.2)	C_1_, C_3_, C_4_	114.8, CH	6.99, d (8.5)	C_1_, C_4_
6	119.1, CH	7.10, d (8.2)	C_2_, C_4_, C_7_	126.9, CH	7.74, dd (8.5, 2.2)	C_2_, C_4_, C_7_
7	165.7, C			164.3, C		
8	150.5, C			150.4, C		
9	122.0, CH	7.21, d (8.6)	C_8_, C_11_, C_13_	122.1, CH	7.20, d (8.6)	C_8_, C_11_, C_13_
10	127.1, CH	7.55, d (8.6)	C_8_, C_12_, C_14_	127.1, CH	7.54, d (8.6)	C_8_, C_12_, C_14_
11	134.7, C			134.7, C		
12	127.1, CH	7.55, d (8.6)	C_8_, C_10_, C_14_	127.1, CH	7.54, d (8.6)	C_8_, C_10_, C_14_
13	122.0, CH	7.21, d (8.6)	C_8_, C_9_, C_11_	122.1, CH	7.20, d (8.6)	C_8_, C_9_, C_11_
14	135.8, CH	6.74, dd (17.7, 10.9)	C_10_, C_11_, C_12_	135.8, CH	6.74, dd (17.6, 10.9)	C_10_, C_11_, C_12_
15	114.3, CH_2_	5.29, dd (10.9, 0.6); 5.82, dd (17.7, 0.6)	C_11_, C_14_	114.3, CH_2_	5.27, dd (10.9, 0.7); 5.82, dd (17.6, 0.7)	C_11_, C_14_
16	35.5, CH_2_	2.98, dd (13.1, 8.4); 3.11, dd (13.1, 5.4)	C_2_, C_17_, C_18_	160.2, CH	8.33, d (1.6)	C_3_
17	52.2, CH	4.25, m	C_18_, C_19_			
18	171.0, C					
19	169.3, C					
20	22.2, CH_3_	1.81, s	C_19_			
21	51.9, CH_3_	3.54, s	C_18_			
NH		8.41, d (7.5)	C_17_, C_19_		9.78, s	C_3_

**Table 2:** Antimicrobial activity of **1**–**5** (MIC in μM).

Microorganisms	Compounds					Gentamicin	Amphotericin B
	1	2	3	4	5		
*E. coli*	41.8	67.1	125.4	73.9	112.4	1.44	
MRSA	116.2	176.5	125.4	110.9	116.5	0.36	
*C. albicans*	116.2	176.5	121.4	104.3	95.7		1.08

## Supplementary Materials

The following are available online at http://www.mdpi.com/1660-3397/16/9/330/s1, Figure S1: Colony picture of strains ZZ745, Figure S2: 16S rDNA sequence of *Streptomyces* sp. ZZ745, Figures S3–S16: NMR, HRESIMS, UV, and IR spectra of bagremycins F (**1**) and G (**2**), Figures S17–S22: NMR spectra of bagremycins A (**3**), B (**4**), and E (**5**), Figure S23: HPLC profile of bagremycin F (**1**) and ethanol crude extract, Figure S24: the analytic result of the ethanol crude extract by HPLC-HRESIMS, Table S1: Sequences producing significant alignments of strain ZZ745, Table S2: NMR data of bagremycins A (**3**), B (**4**), and E (**5**) (in DMSO-*d*_6_).
