# Supplementary material for: New Antibacterial Bagremycins F and G from the Marine-Derived Streptomyces sp. ZZ745
Source: Mar Drugs. 2018 Sep 12;16(9):330. doi: 10.3390/md16090330 (PMC6163353; doi:10.3390/md16090330)

Supplementary Materials for

New Antibacterial Bagremycins F and G from Marine-derived *Streptomyces* sp. ZZ745

Di Zhang [1,†], Chenyan Shu [2,†], Xiaoyuan Lian [2,*] and Zhizhen Zhang, [1,*]

[1] Ocean College, Zhoushan Campus, Zhejiang University, Zhoushan 316021, China; zhangdi163.love@163.com

[2] College of Pharmaceutical Sciences, Zhejiang University, Hangzhou 310058, China; 21519023@zju.edu.cn

* Correspondence: xylian@zju.edu.cn (X.L.); zzhang88@zju.edu.cn (Z.Z.); Tel.: +86-135-7547-6388 (X.L.); +86-136-7585-9706 (Z.Z.)

† These authors contributed equal to this work.

CONTENT

Table S_1. Sequences producing significant alignments of strain ZZ745……………..…………..…3

Table S_2. ^{13}C and ^{1}H NMR data of bagremycins A (**3**), B (**4**), and E (**5**) (in DMSO-d_6)……....4

Figure S_1. Colony picture of strains ZZ745………………………..5

Figure S_2. 16S rDNA sequence of *Streptomyces* sp. ZZ745…………………………………….5

Figure S_3. ^{1}H NMR spectrum of bagremycin F (**1**)………………………………………...6

Figure S_4. ^{13}C NMR spectrum of bagremycin F (**1**)………………………………………...7

Figure S_5. HSQC spectrum of bagremycin F (**1**)……………………………….……………...8

Figure S_6. HMBC spectrum of bagremycin F (**1**)…………………….………..……..………..9

Figure S_7. HRESIMS spectrum of bagremycin F (**1**)……………….……………………..10

Figure S_8. UV spectrum of bagremycin F (**1**)…………………….…………………………..10

Figure S_9. IR spectrum of bagremycin F (**1**)……………………………..…………………..10

Figure S_{10}. ^{1}H NMR spectra of bagremycin G (**2**)……………………………………….....11

Figure S_{11}. ^{13}C NMR spectrum of bagremycin G (**2**)…….…………………………..……...12

Figure S_{12}. HSQC spectrum of bagremycin G (**2**)…………………………..……..…...13

Figure S_{13}. HMBC spectrum of bagremycin G (**2**)…….…………..……..……..…...14

Figure S_{14}. HRESIMS spectrum of bagremycin G (**2**)……………………………………15

Figure S_{15}. UV spectrum of bagremycin G (**2**)……………………………………………15

Figure S_{16}. IR spectrum of bagremycin G (**2**)……………………………………………15

Figure S_{17}. ^{1}H NMR spectrum of bagremycin A (**3**)………………………………………16

Figure S_{18}. ^{13}C NMR spectrum of bagremycin A (**3**)……………….………….……….17

Figure S_{19}. ^{1}H NMR spectrum of bagremycin B (**4**)……………………………………..18

Figure S_{20}. ^{13}C NMR spectrum of bagremycin B (**4**)…………….……………………..…19

Figure S_{21}. ^{1}H NMR spectrum of bagremycin E (**5**)………………………………………20

Figure S_{22}. ^{13}C NMR spectrum of bagremycin B (**5**)………………..……..………………21

Figure S_{23}. HPLC profile of bagremycin F (**1**) and ethanol crude extract……………………22

Figure S_{24}. The analytic result of the ethanol crude extract by HPLC-HRESIMS…...............23

Table S_1. Sequences producing significant alignments of strain ZZ745

Accession	Description	Max score	Total score	Query coverage	Evalue	Ident
KC306504.1	*Streptomyces* sp. NEAU-YX9 16S ribosomal RNA gene, partial sequence	2734	2734	97%	0.0	99%
JX047057.1	*Streptomyces* sp. OAct 127 16S ribosomal RNA gene, partial sequence	2730	2730	96%	0.0	99%
KU877596.1	*Streptomyces filipinensis* strain WJA67 16S ribosomal RNA gene, partial sequence	2689	2689	94%	0.0	100%
KU877593.1	*Streptomyces filipinensis* strain WJA60 16S ribosomal RNA gene, partial sequence	2689	2689	94%	0.0	100%
KU877590.1	*Streptomyces filipinensis* strain WJA55 16S ribosomal RNA gene, partial sequence	2687	2687	94%	0.0	100%
LC381941.1	*Streptomyces* sp. MR 602 gene for 16S ribosomal RNA, partial sequence	2673	2673	96%	0.0	99%

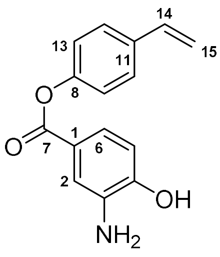

3

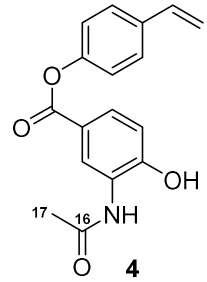

4

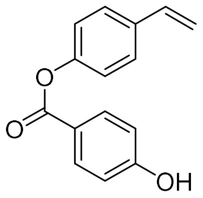

5

Table S2. NMR data of bagremycins A (**3**), B (**4**), and E (**5**) (in DMSO-d_6)

No.	**3**		**4**		**5**	
	^{13}C, type	^{1}H (*J* in Hz)	^{13}C, type	^{1}H (*J*, Hz)	^{13}C, type	^{1}H (*J*, Hz)
1	119.2, C	–	119.1, C	–	119.0, C	–
2	114.9, CH	7.37, d (2.0)	123.8, CH	8.64, d (2.0)	132.2, CH	7.97, d (8.8)
3	137.0, C	–	127.0, C	–	115.7, CH	6.91, d (8.8)
4	149.9, C	–	153.0, C	–	163.0, C	–
5	113.8, CH	6.76, d (8.3)	115.3, CH	7.01, d (8.5)	115.7, CH	6.91, d (8.8)
6	119.8, CH	7.25, dd (8.3, 2.0)	126.6, CH	7.74, dd (8.5, 2.0)	132.2, CH	7.97, d (8.8)
7	164.9, C	–	164.3, C	–	164.3, C	–
8	150.6, C	–	150.4, C	–	150.4, C	–
9	122.2, CH	7.17, d (8.5)	122.1, CH	7.20, d (8.6)	122.2, CH	7.21, d (8.6)
10	127.1, CH	7.53, d (8.5)	127.2, CH	7.54, d (8.6)	127.1, CH	7.53, d (8.6)
11	134.6, C	–	134.8, C	–	134.7, C	–
12	127.1, CH	7.53, d (8.5)	127.2, CH	7.54, d (8.6)	127.1, CH	7.52, d (8.6)
13	122.2, CH	7.17, d (8.5)	122.1, CH	7.20, d (8.6)	122.2, CH	7.21, d (8.6)
14	135.8, CH	6.73, dd (17.6, 11.0)	135.8, CH	6.74, dd (17.7, 11.0)	135.8, CH	6.73, dd (17.7, 11.0)
15	114.3, CH_2	5.26, dd (11.0, 0.7); 5.81, dd (17.6, 0.7)	114.4, CH_2	5.26, dd (11.0, 0.6); 5.82, dd (17.7, 0.6)	114.4, CH_2	5.26, dd (11.0, 0.7); 5.81, dd (17.7, 0.7)
16			169.1, C	–		
17			23.7, CH_3	2.12, s		
NH				9.38, s		

Figure S_1. Colony picture of strains ZZ745

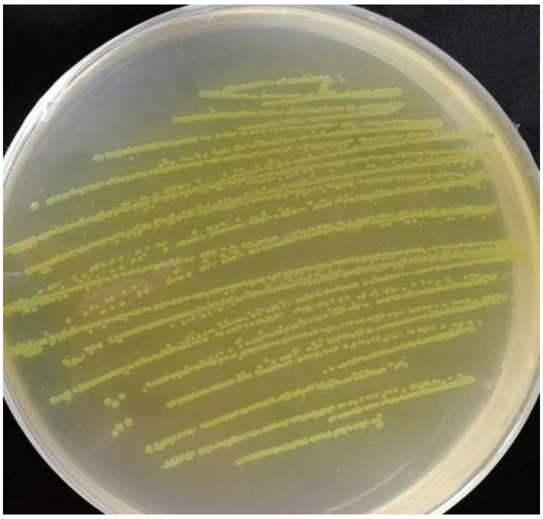

Figure S_2. 16S rDNA sequence of *Streptomyces* sp. ZZ745

CGGCCTATCGCGTGTCGCCCTTAGAGTTTGATCCTGGCTCAGGACGAACGCTGGCGGCGTGCT
TAACACATGCAAGTCGAACGATGAAGCCCTTCGGGGTGGATTAGTGGCGAACGGGTGAGTAA
CACGTGGGCAATCTGCCCTTCACTCTGGGACAAGCCCTGGAAACGGGGTCTAATACCGGATAT
GAGCCTCCCAGGCATCTGGGAGGCTGTAAAGCTCCGGCGGTGAAGGATGAGCCCGCGGCCTA
TCAGCTTGTTGGTGAGGTAATGGCTCACCAAGGCGACGACGGGTAGCCGGCCTGAGAGGGCG
ACCGGCCACACTGGGACTGAGACACGGCCCAGACTCCTACGGGAGGCAGCAGTGGGGAATAT
TGCACAATGGGCGAAAGCCTGATGCAGCGACGCCGCGTGAGGGATGACGGCCTTCGGGTTGT
AAACCTCTTTCAGCAGGGAAGAAGCGAGAGTGACGGTACCTGCAGAAGAAGCGCCGGCTAA
CTACGTGCCAGCAGCCGCGGTAATACGTAGGGCGCAAGCGTTGTCCGGAATTATTGGGCGTAA
AGAGCTCGTAGGCGGCTTGTCACGTCGATTGTGAAAGCTCGGGGCTTAACCCCGAGTCTGCA
GTCGATACGGGCTAGCTAGAGTGTGGTAGGGGAGATCGGAATTCCTGGTGTAGCGGTGAAATG
CGCAGATATCAGGAGGAACACCGGTGGCGAAGGCGGATCTCTGGGCCATTACTGACGCTGAG
GAGCGAAAGCGTGGGGAGCGAACAGGATTAGATACCCTGGTAGTCCACGCCGTAAACGGTGG
GAACTAGGTGTTGGCGACATTCCACGTCGTCGGTGCCGCAGCTAACGCATTAAGTTCCCCGCC
TGGGGAGTACGGCCGCAAGGCTAAAACTCAAAGGAATTGACGGGGGCCCGCACAAGCGGCG
GAGCATGTGGCTTAATTCGACGCAACGCGAAGAACCTTACCAAGGCTTGACATACACCGGAA
AGCATTAGAGATAGTGCCCCCCTTGTGGTCGGTGTACAGGTGGTGCATGGCTGTCGTCAGCTC
GTGTCGTGAGATGTTGGGTTAAGTCCCGCAACGAGCGCAACCCTTGTTCTGTGTTGCCAGCAT
GCCCTTCGGGGTGATGGGGACTCACAGGAGACCGCCGGGGTCAACTCGGAGGAAGGTGGGG
ACGACGTCAAGTCATCATGCCCCTTATGTCTTGGGCTGCACACGTGCTACAATGGCCGGTACA
ATGAGCTGCGATACCGTGAGGTGGAGCGAATCTCAAAAAGCCGGTCTCAGTTCGGATTGGGG
TCTGCAACTCGACCCCATGAAGTCGGAGTCGCTAGTAATCGCAGATCAGCATTGCTGCGGTGA
ATACGTTCCCGGGCCTTGTACACACCGCCCGTCACGTCACGAAAGTCGGTAACACCCGAAGC
CGGTGGCCCAACCCCTTGTGGGAGGGAGCTGTCGAAGGTGGGACTGGCGATTGGGACGAAG
TCGTAACAAGGTAACCAAGGGCGACACGCGATGGAGCACT

Figure S3. ^{1}H NMR spectrum of bagremycin F (**1**, 500 MHz, in DMSO-d_6)

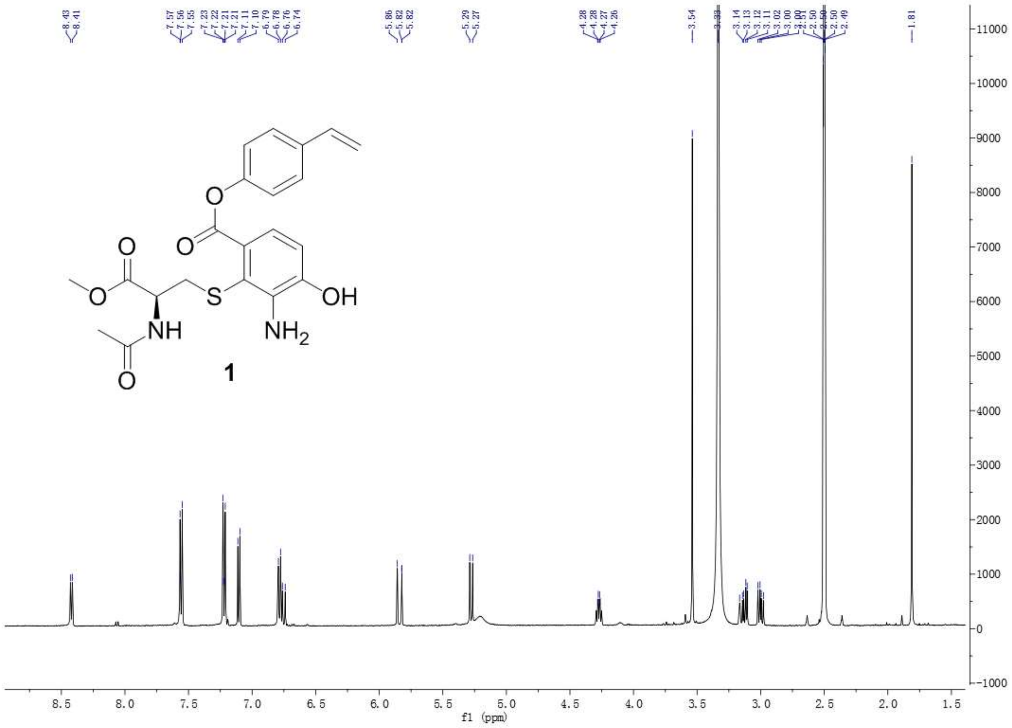

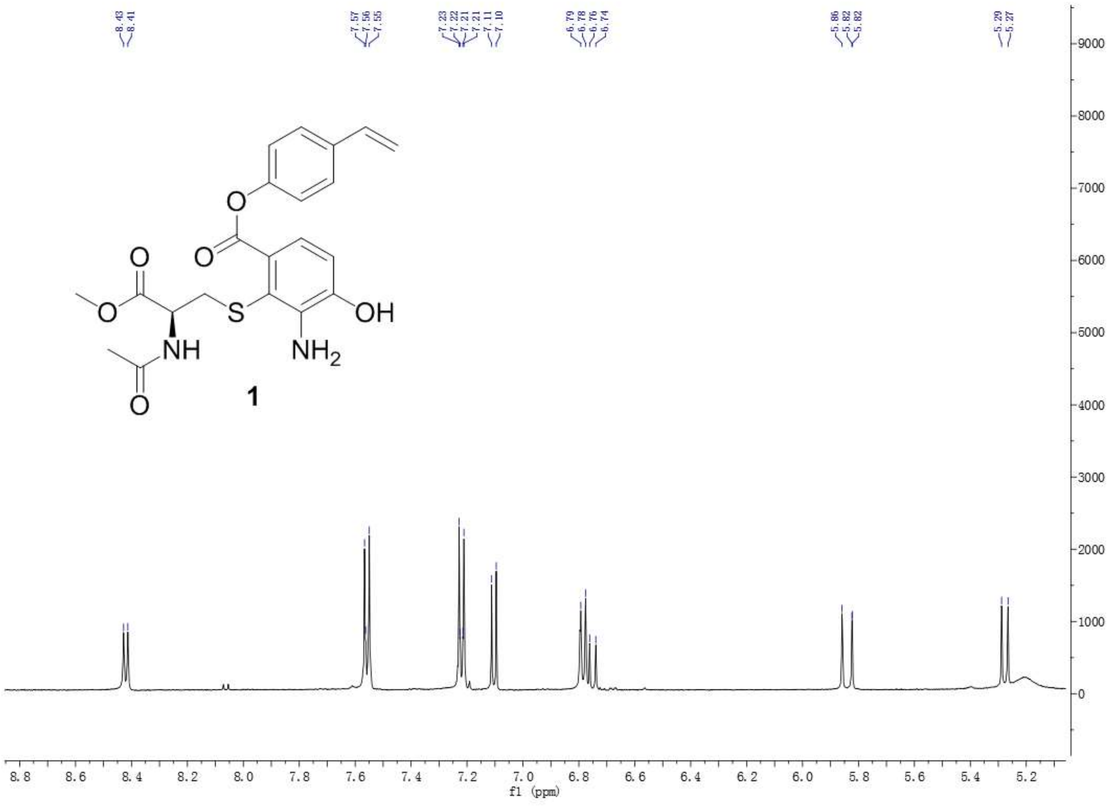

Figure S4. ^{13}C NMR spectrum of bagremycin F (**1**, 125 MHz, in DMSO-d_6)

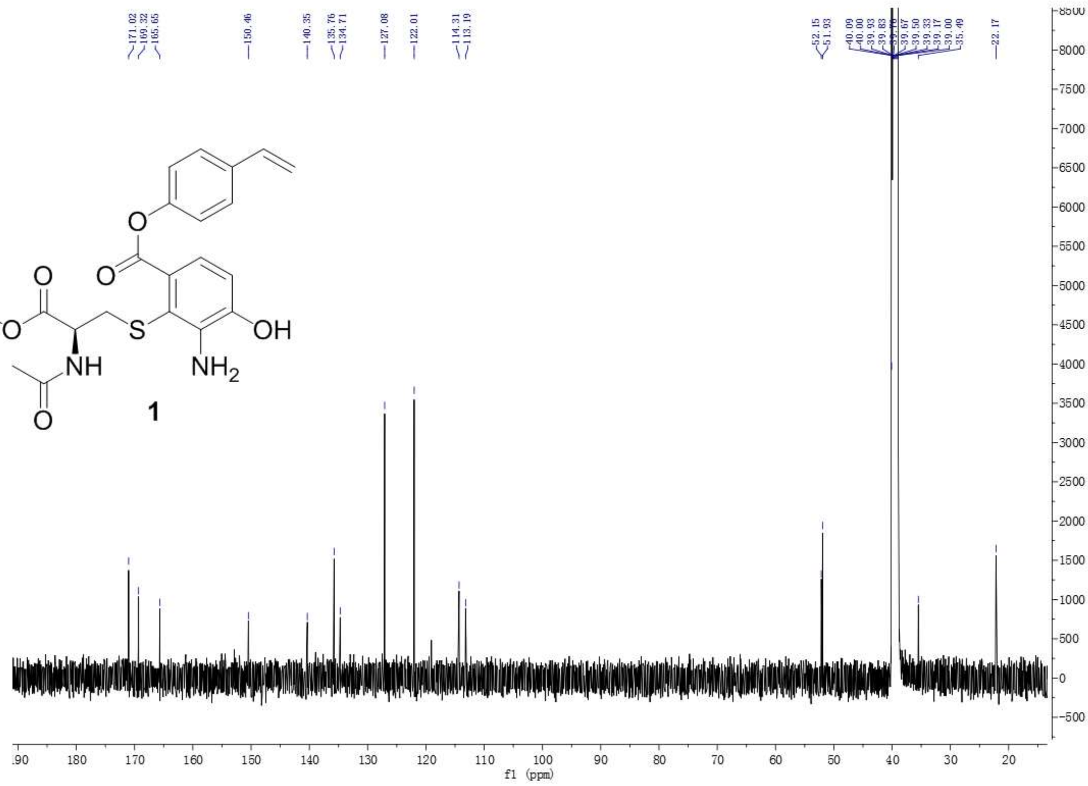

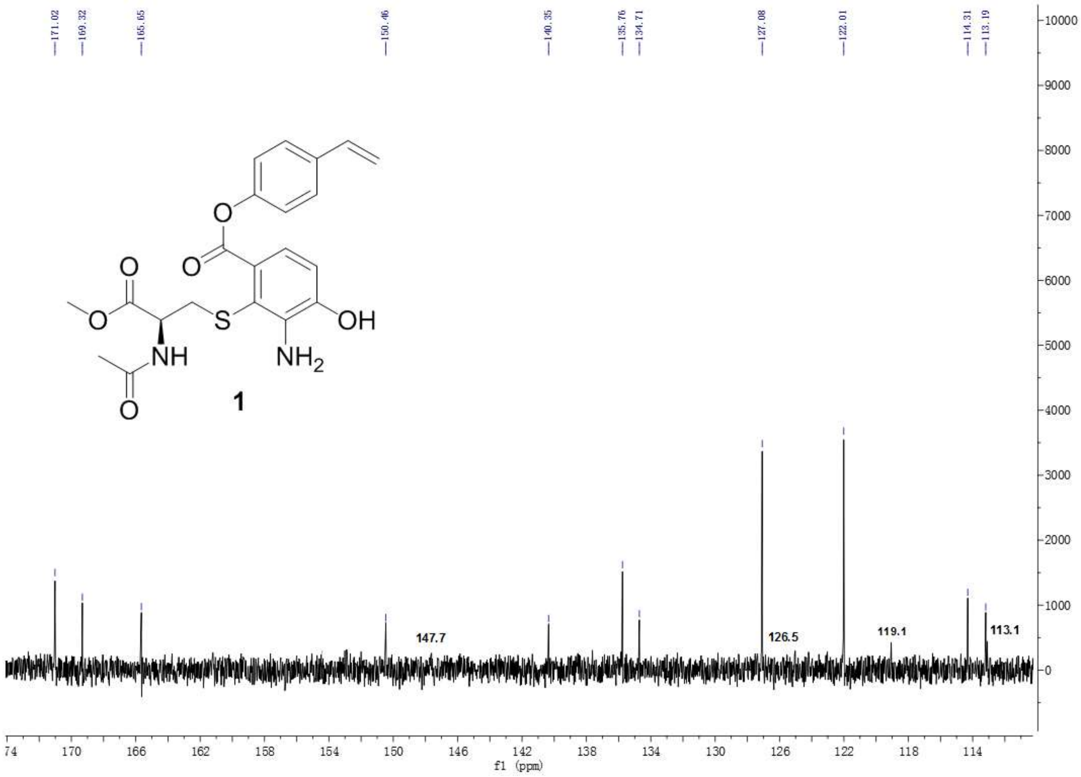

Figure S5. HSQC spectrum of bagremycin F (**1**, in DMSO-d_6)

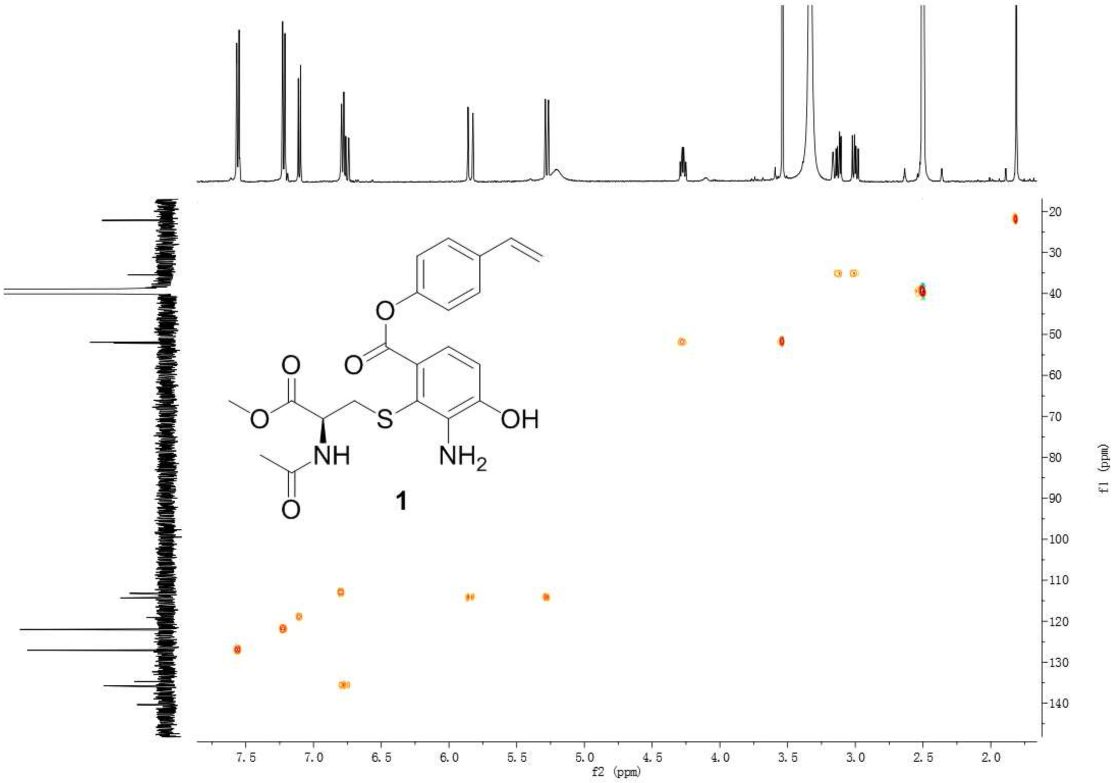

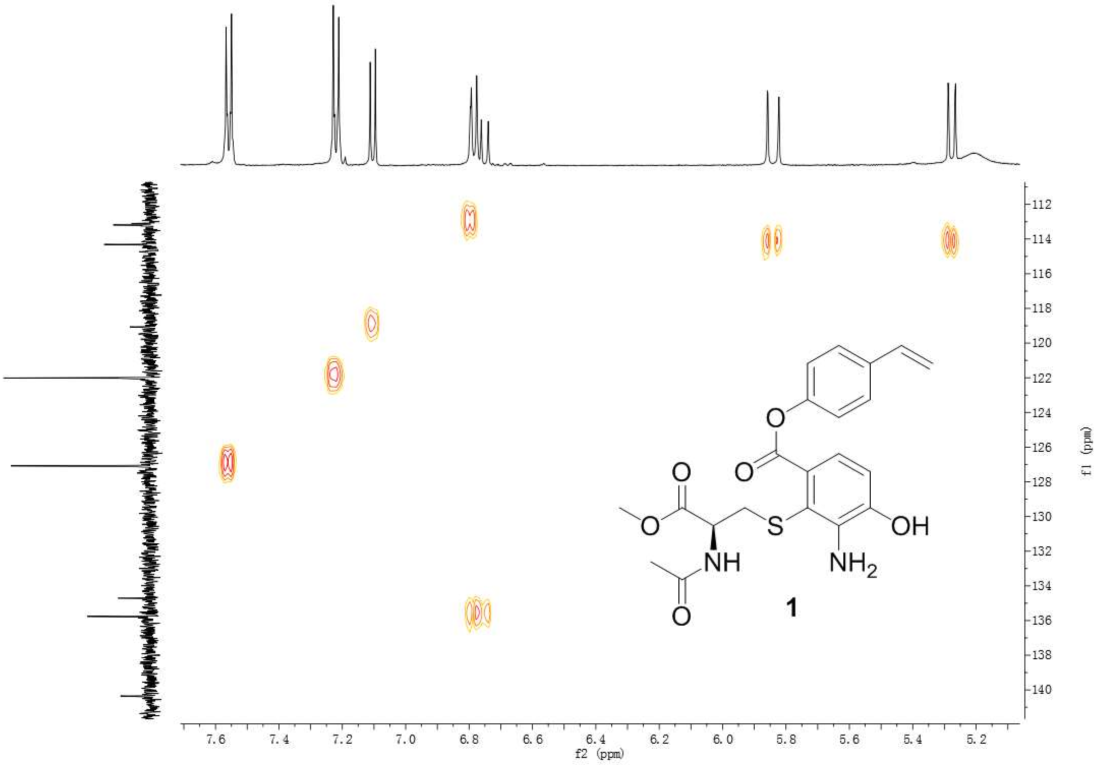

Figure S6. HMBC spectrum of bagremycin F (**1**, in DMSO-d_6)

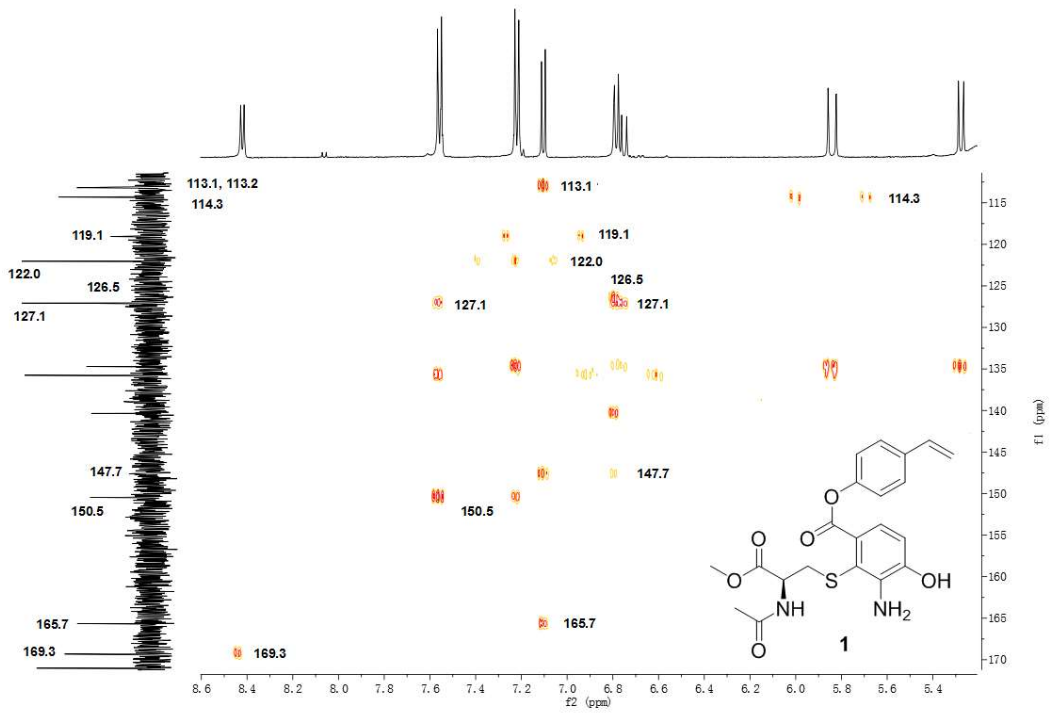

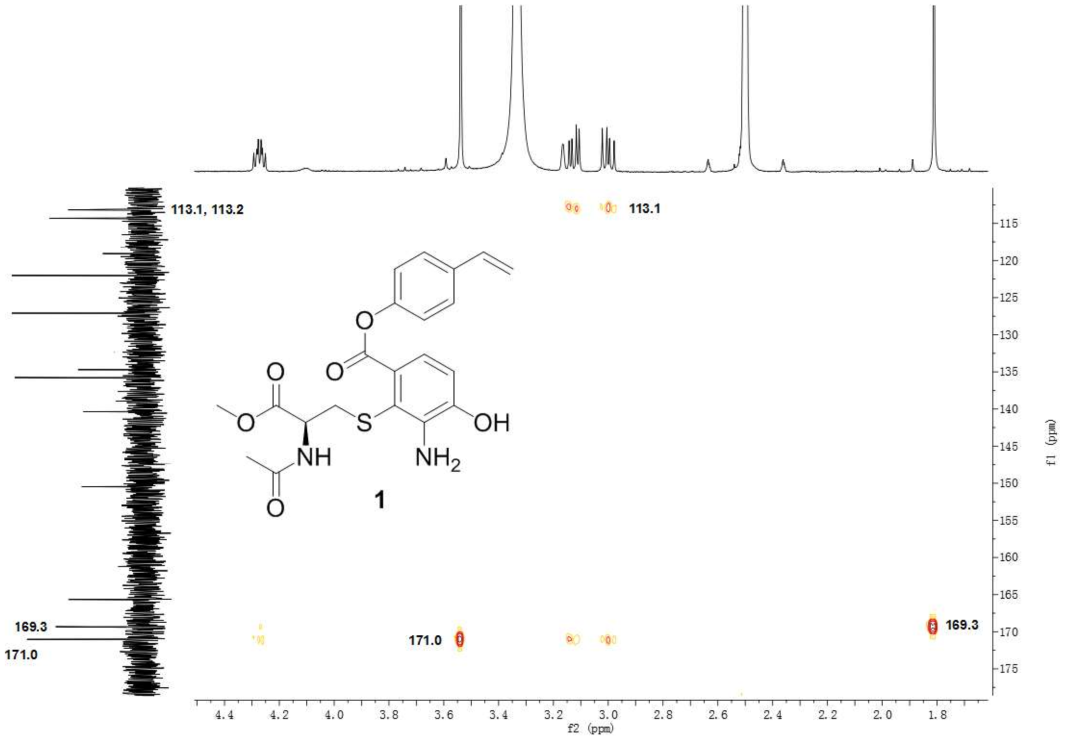

Figure S7. HRESIMS spectrum of bagremycin F (**1**)

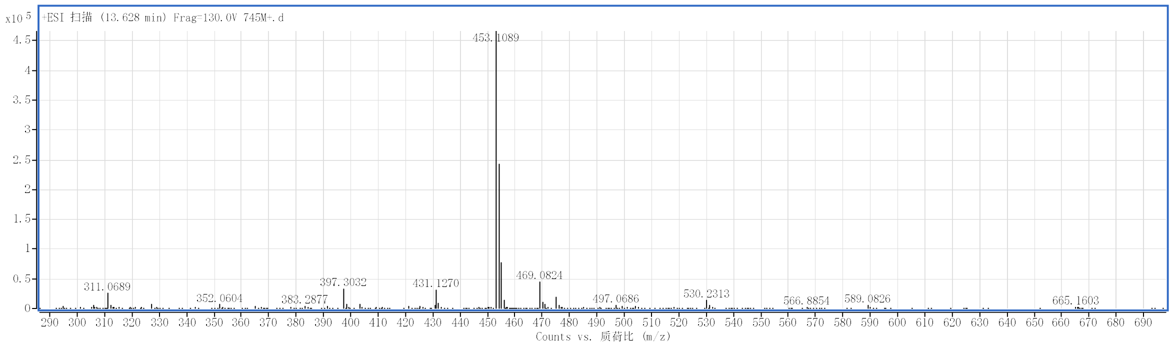

Figure S8. UV spectrum of bagremycin F (**1**)

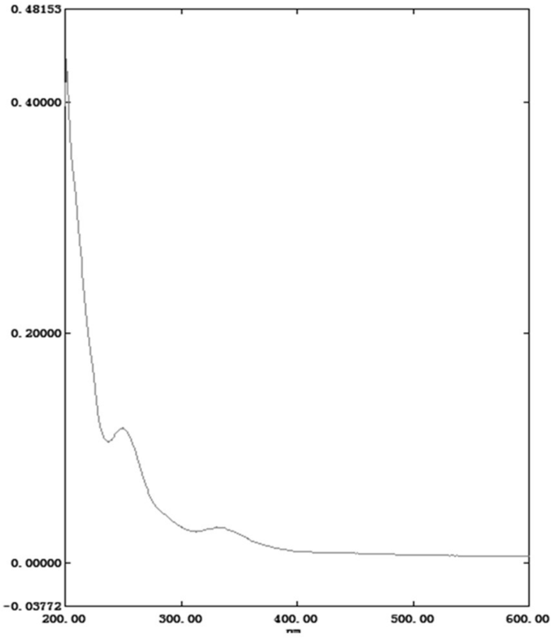

Figure S9. IR spectrum of bagremycin F (**1**)

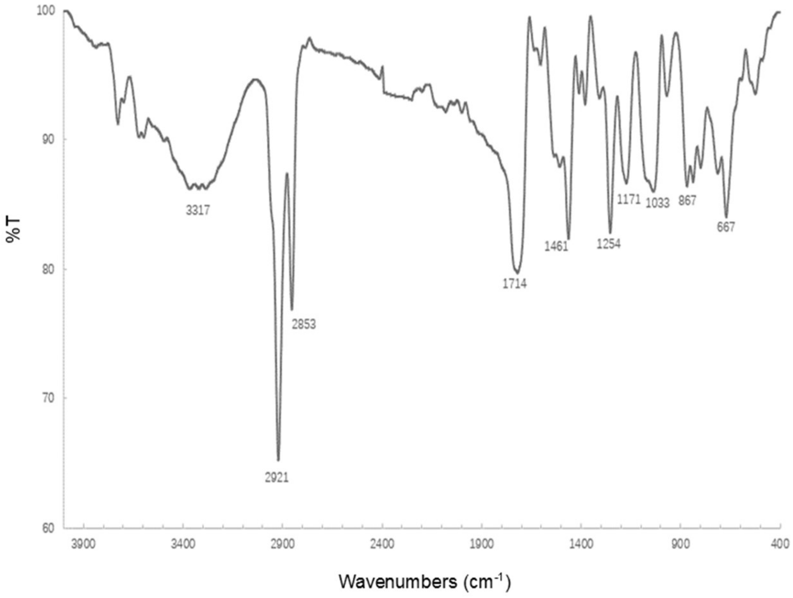

Figure S10. ^{1}H NMR spectrum of bagremycin G (**2**, 500 MHz, in DMSO-d_6)

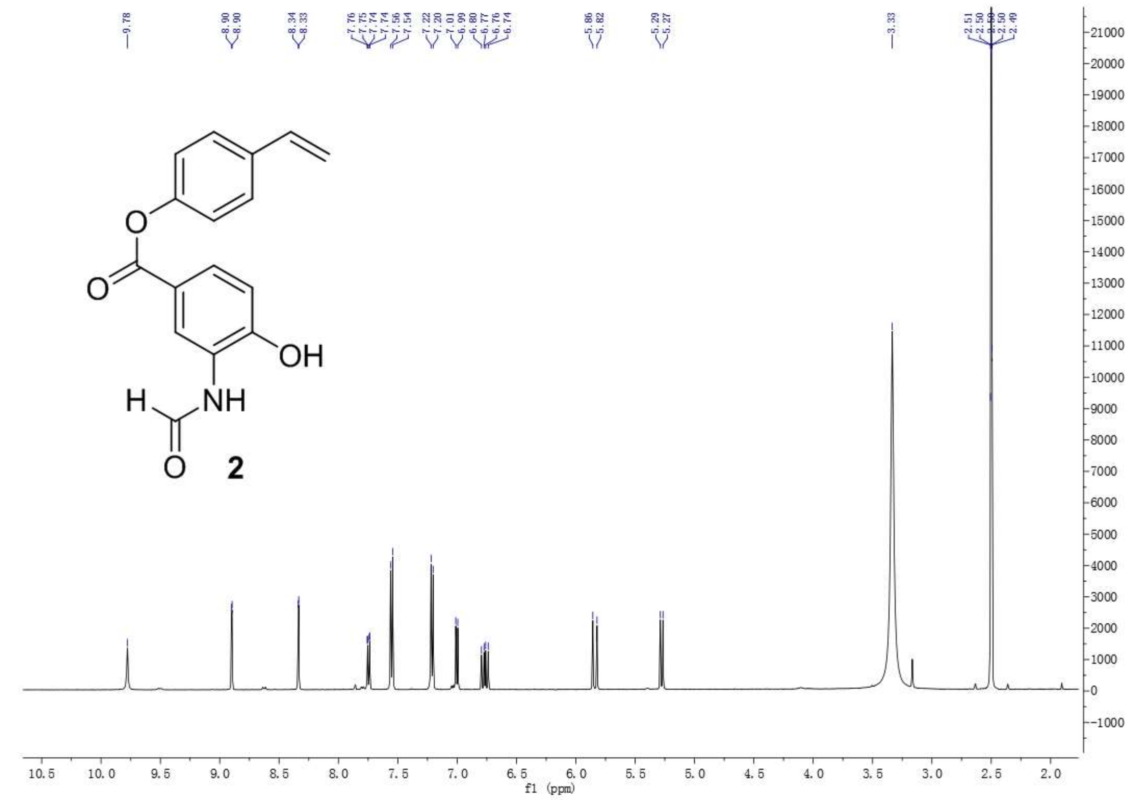

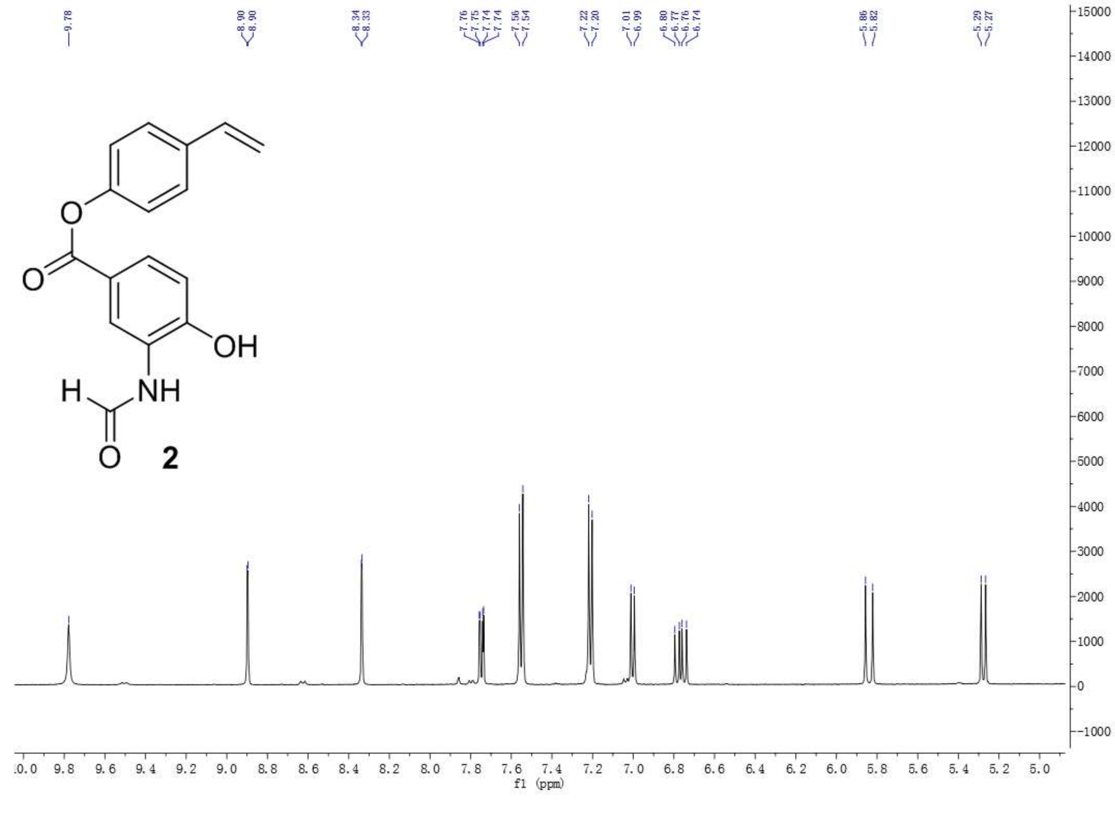

Figure S11. ^{13}C NMR spectrum of bagremycin G (**2**, 125 MHz, in DMSO-d_6)

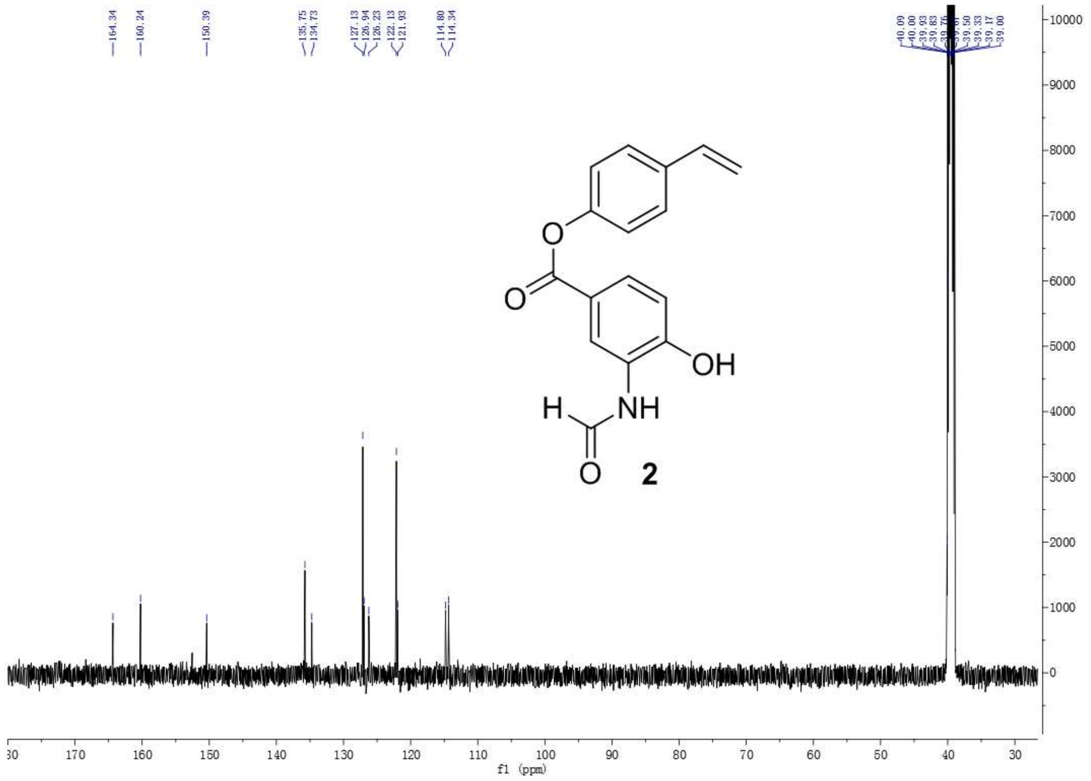

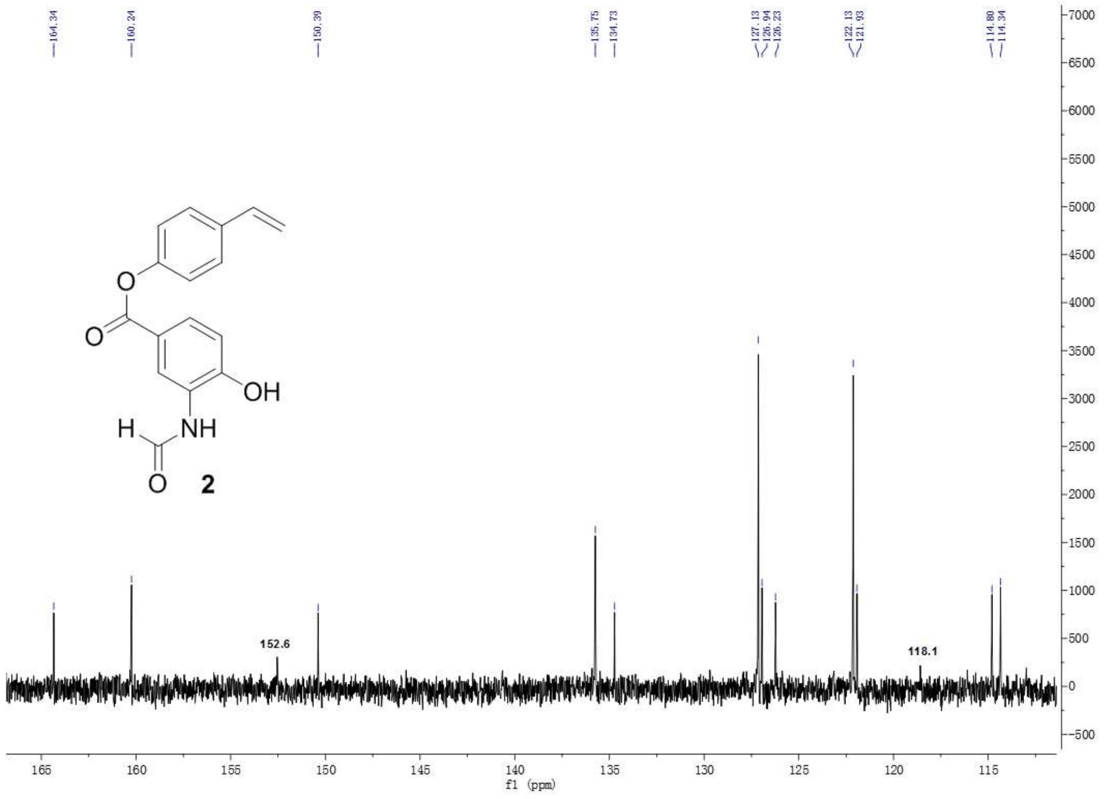

Figure S12. HSQC spectrum of bagremycin G (**2**, in DMSO-d_6)

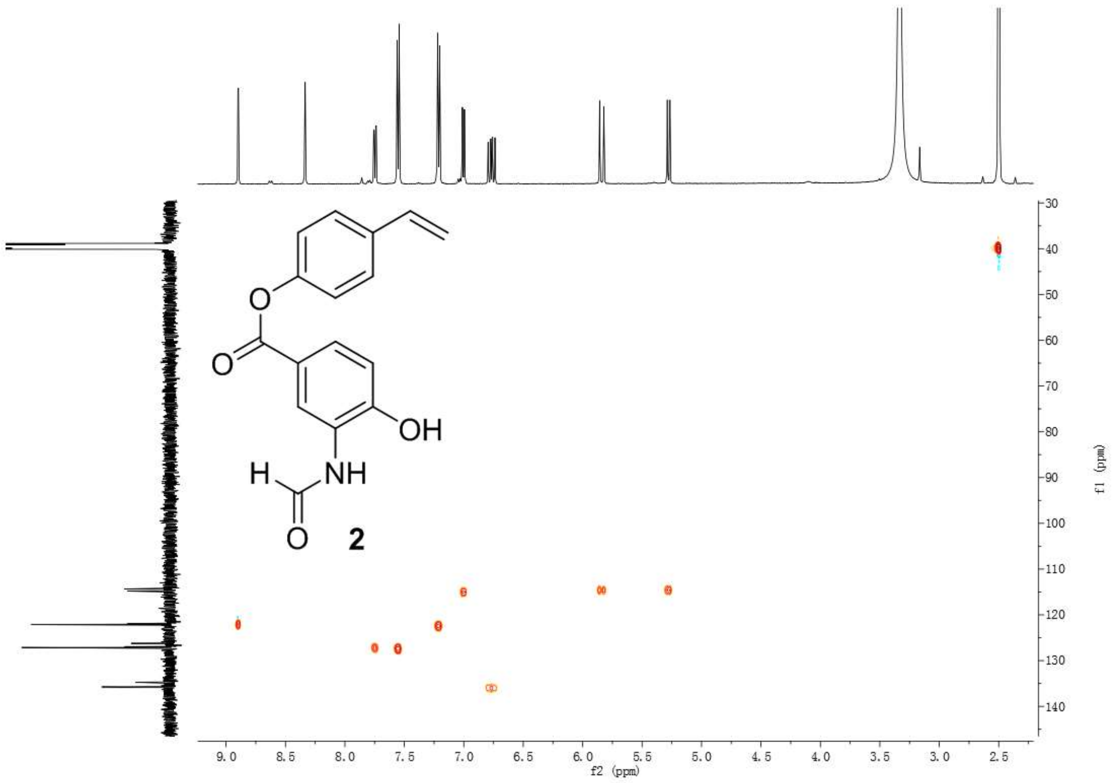

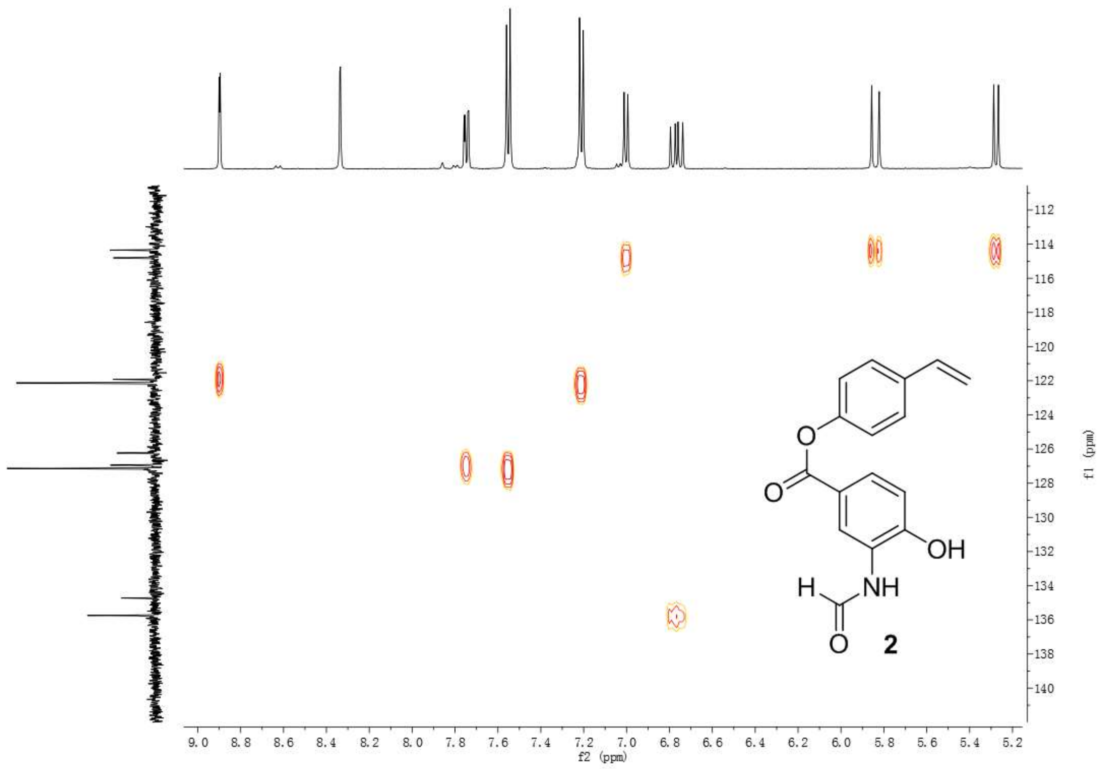

Figure S13. HMBC spectrum of bagremycin G (**2**, in DMSO-d_6)

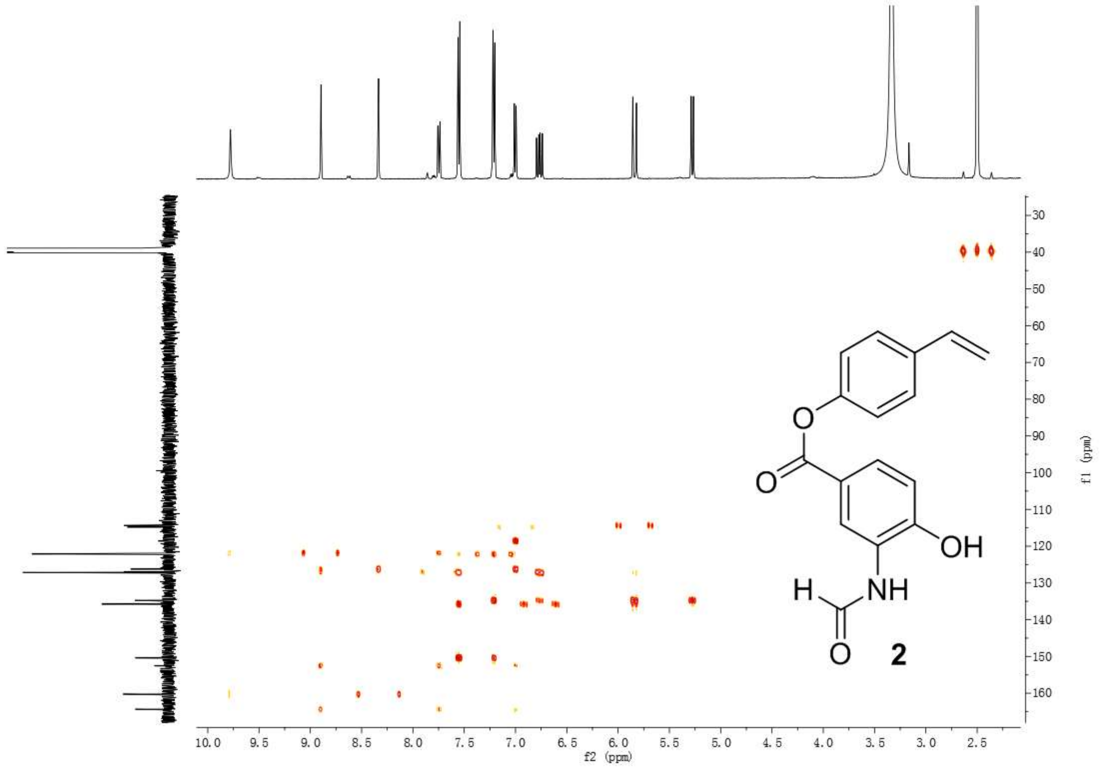

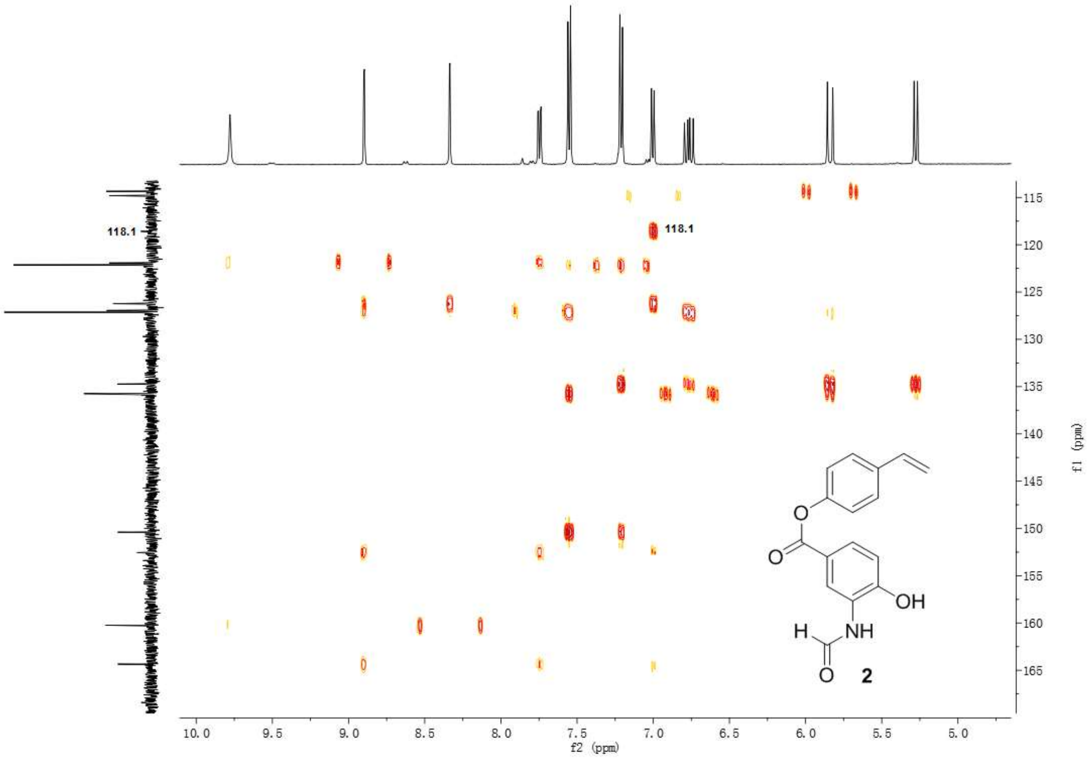

Figure S14. HRESIMS spectrum of bagremycin G (**2**)

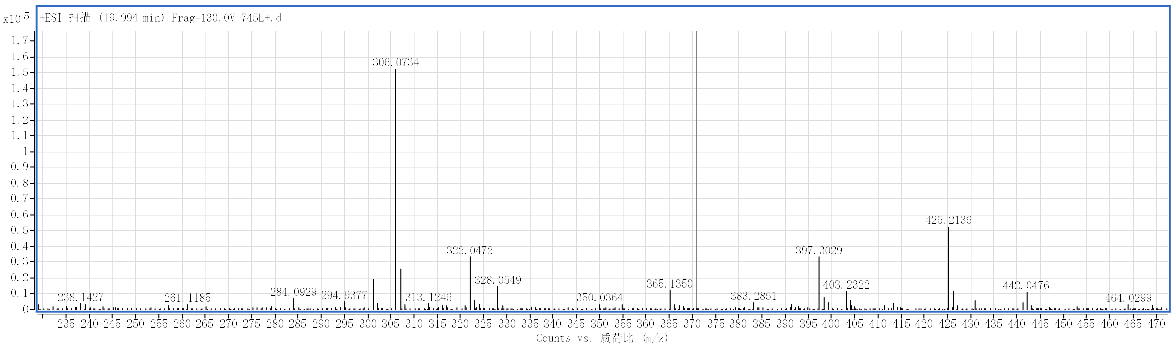

Figure S15. UV spectrum of bagremycin G (**2**)

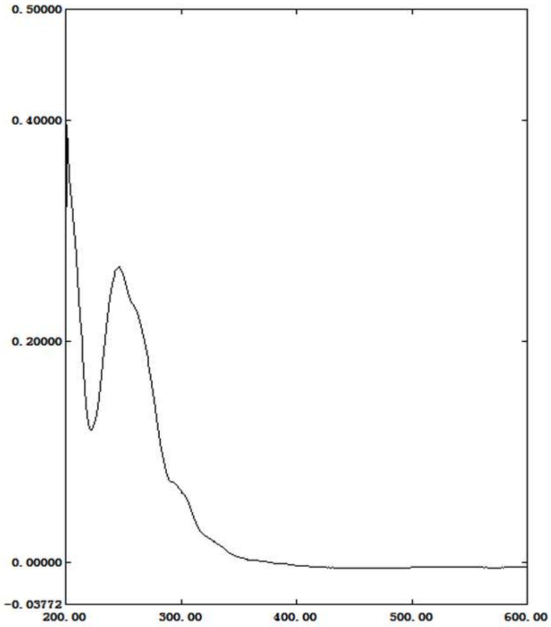

Figure S16. IR spectrum of bagremycin G (**2**)

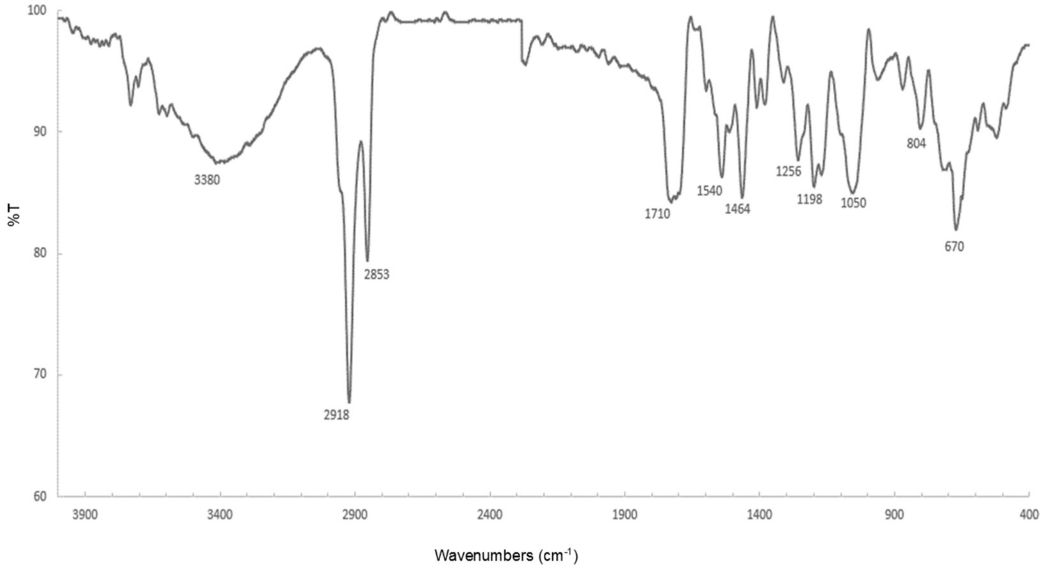

Figure S17. ^{1}H NMR spectrum of bagremycin A (**3**, 500 MHz, in DMSO-d_6)

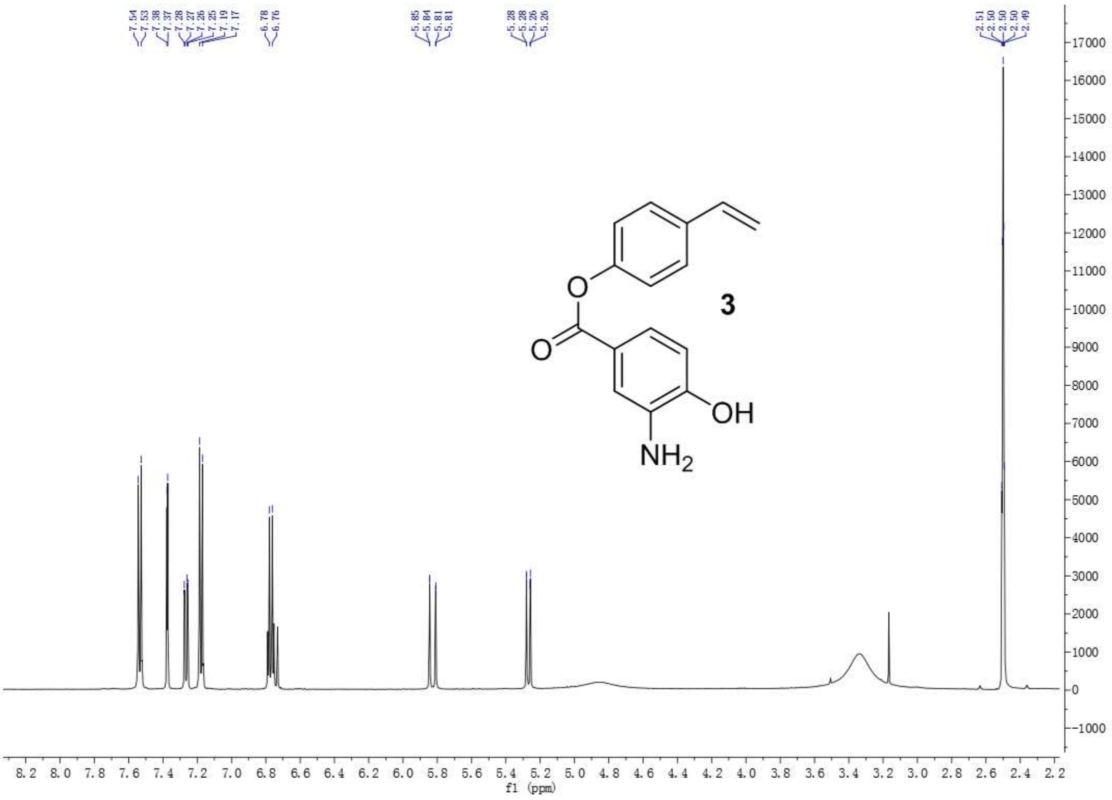

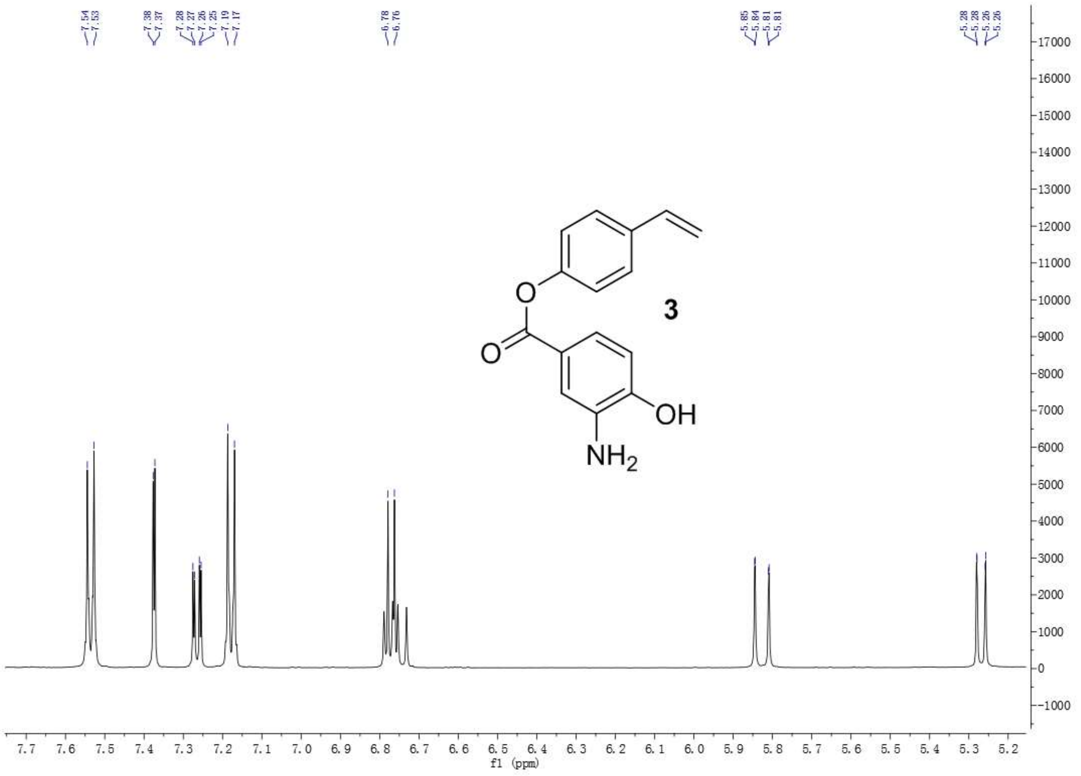

Figure S18. ^{13}C NMR spectrum of bagremycin A (**3**, 125 MHz, in DMSO-d_6)

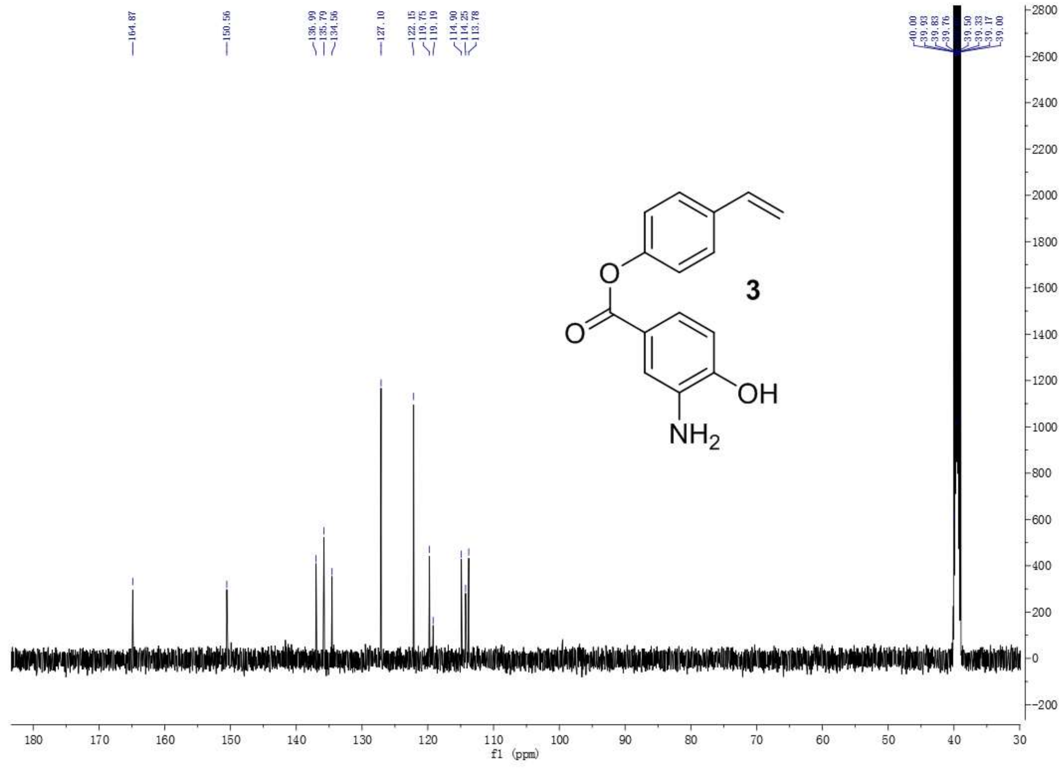

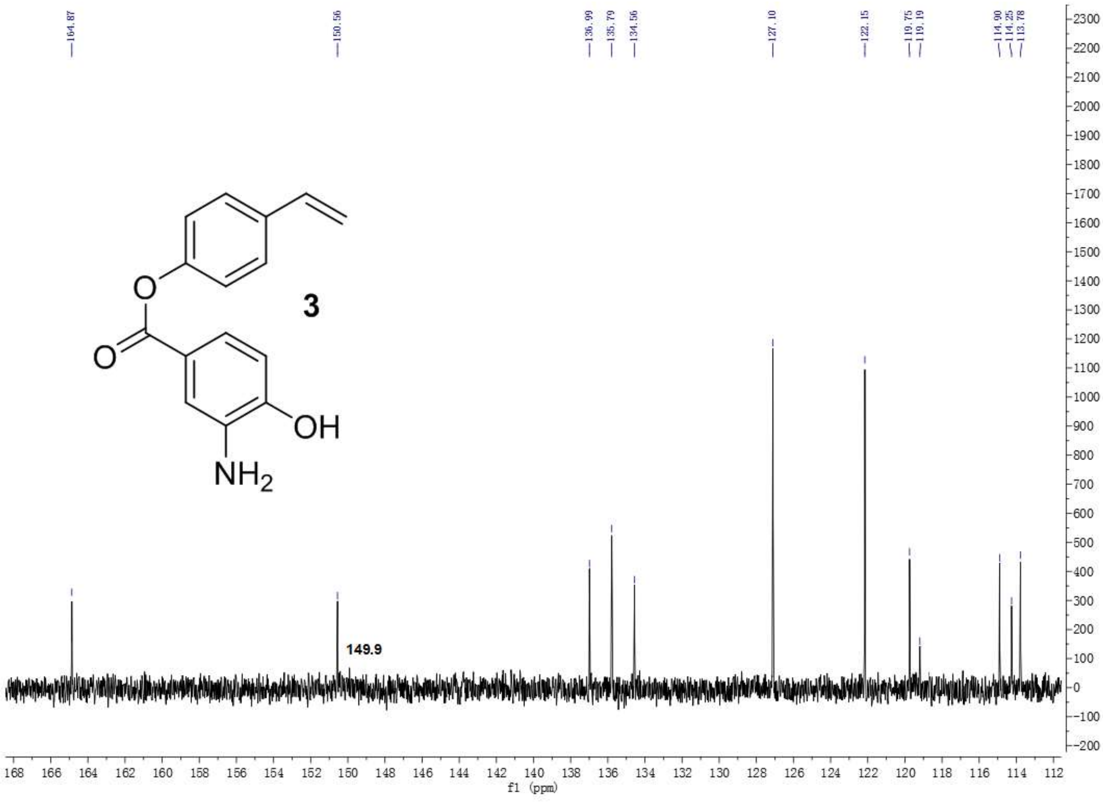

Figure S19. ^{1}H NMR spectrum of bagremycin B (**4**, 500 MHz, in DMSO-d_6)

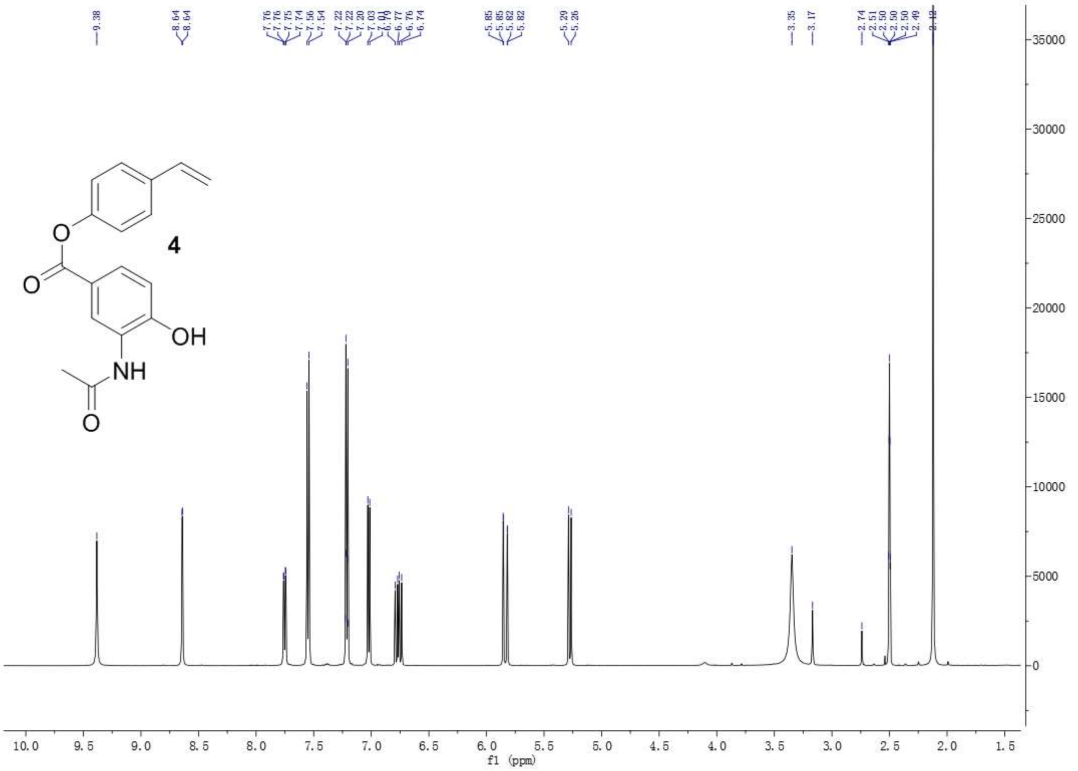

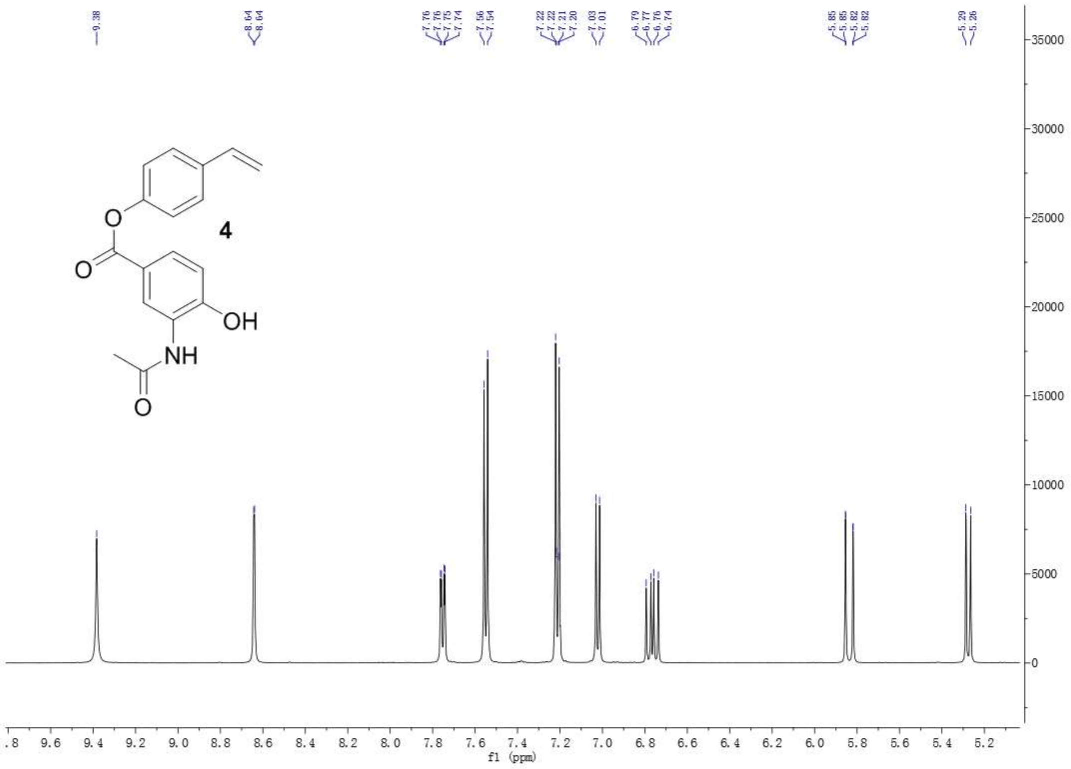

Figure S20. ^{13}C NMR spectrum of bagremycin B (**4**, 125 MHz, in DMSO-d_6)

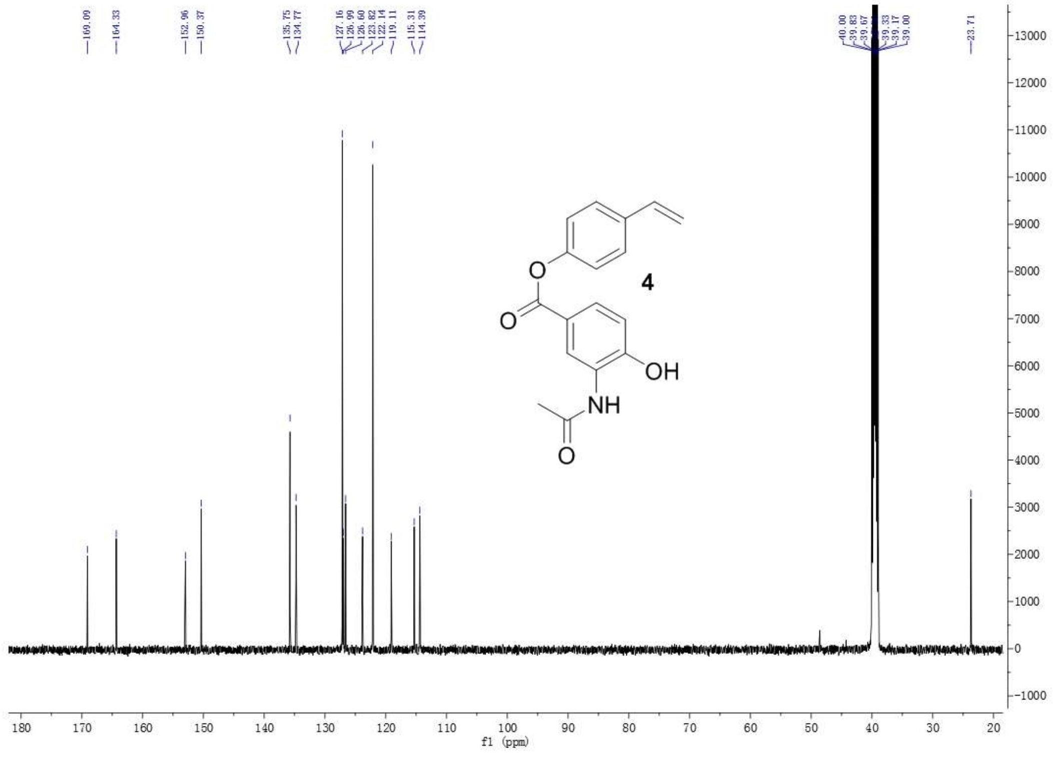

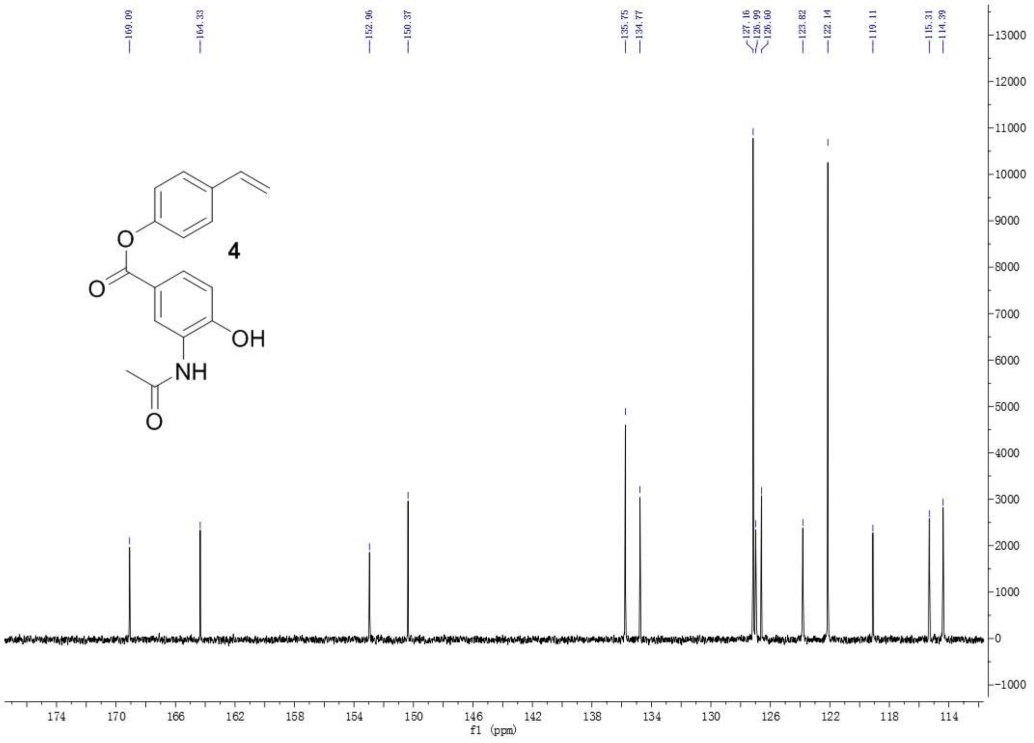

Figure S21. ^{1}H NMR spectrum of bagremycin E (**5**, 500 MHz, in DMSO-d_6)

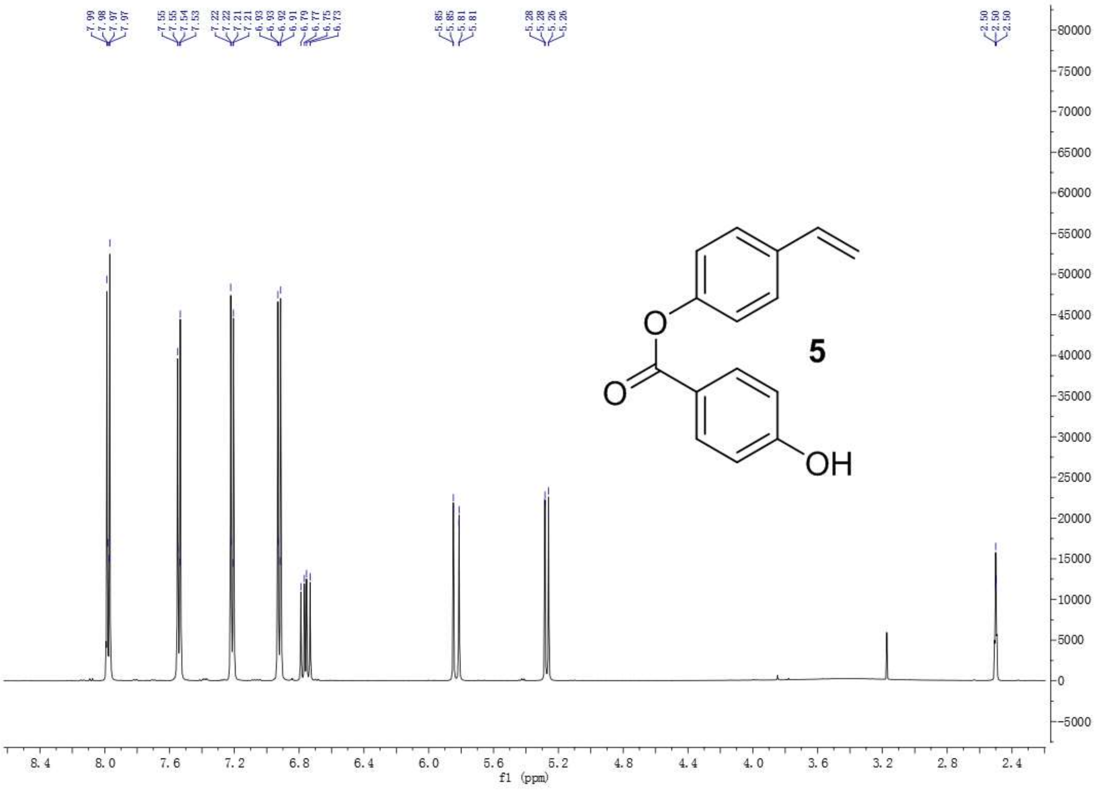

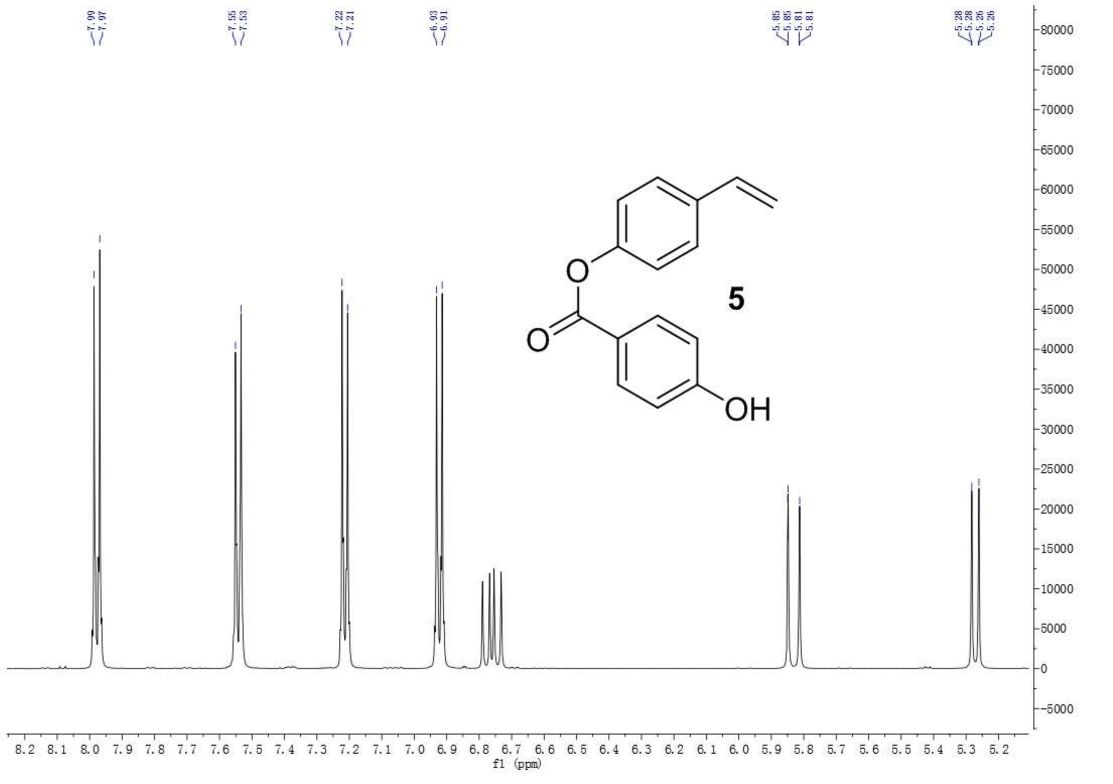

Figure S22. ^{13}C NMR spectrum of bagremycin E (**5**, 125 MHz, in DMSO-d_6)

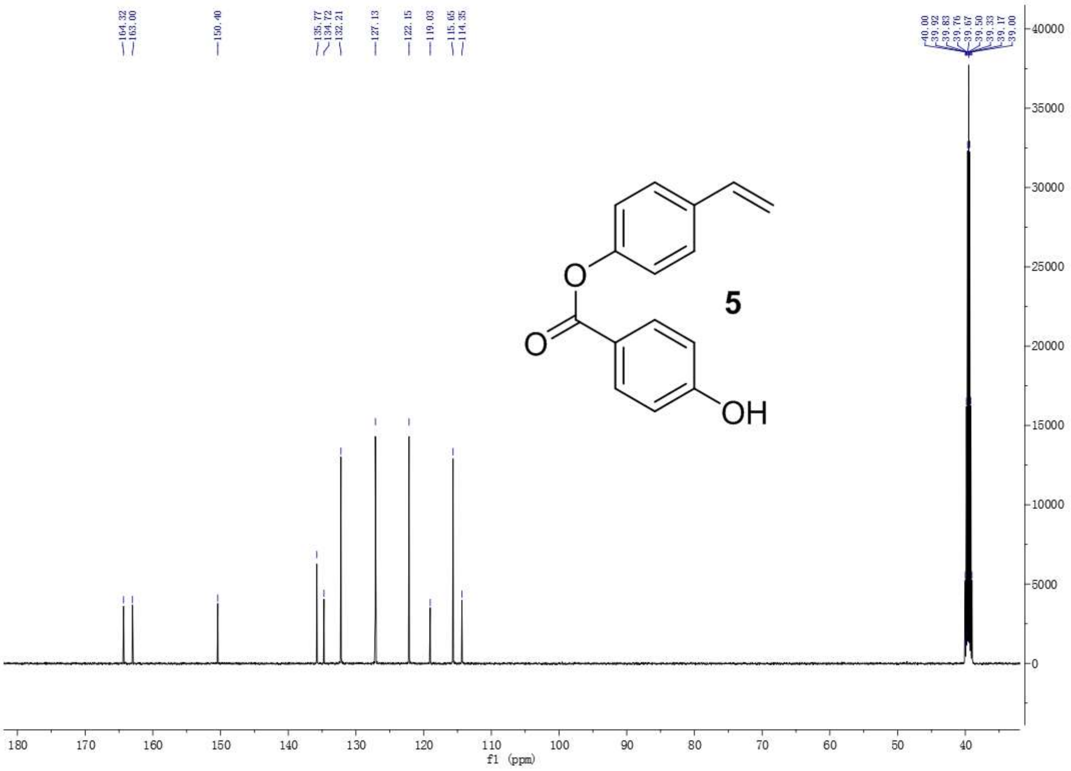

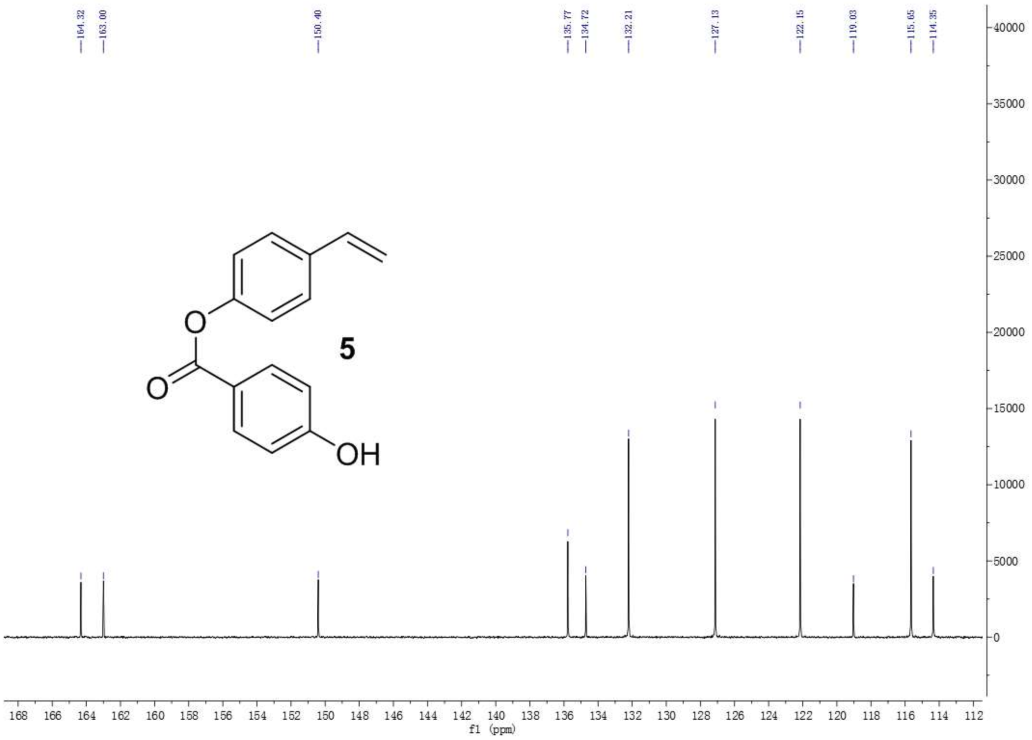

Figure S23. HPLC profile of bagremycin F (**1**) and ethanol crude extract (A: compound **1** + crude extract; B: crude extract; C: compound **1**). The separation of compounds was conducted on a ZORBAX SB-C18 HPLC colunm (250 × 4.6 mm, 5 μm) on the conditions of UV detection at 256 nm and flow rate at 1.0 mL/min. Acetonitrile and water were used as mobile phases A and B, respectively. The binary gradient program was 0.0–40.0 min with 20–100% A, 40.1–48.0 min with 100% A, and 48.1–60.0 min with 20% A.

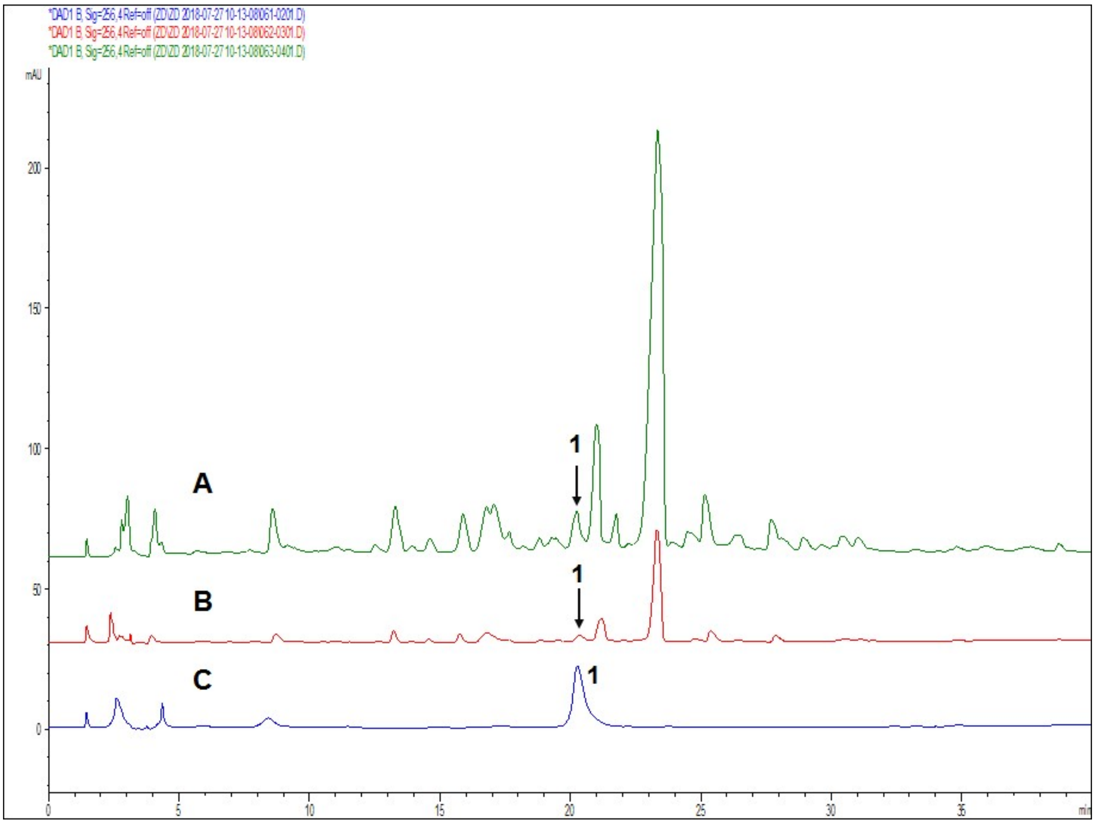

Figure S24. The analytic result of the ethanol crude extract by HPLC-HRESIMS. The separation was conducted on an Agilent Poroshell 120 EC-18 column (50 × 4.6 mm, 2.7 μm) with UV detection at 256 nm and flow rate at 0.6 mL/min. Acetonitrile and water were employed as mobile phases A and B, respectively. The binary gradient program was 0.0–10.0 min with 10–50% A, 10.1–15.0 min with 100% A, and 15.1–18.0 min with 10% A. Bagremycin F (**1**) was detected at t_R 12.44 min with a $[M + Na]^+$ ion at *m/z* 453.1092 (calcd for $C_{21}H_{22}N_2NaO_6S$, 453.1096).

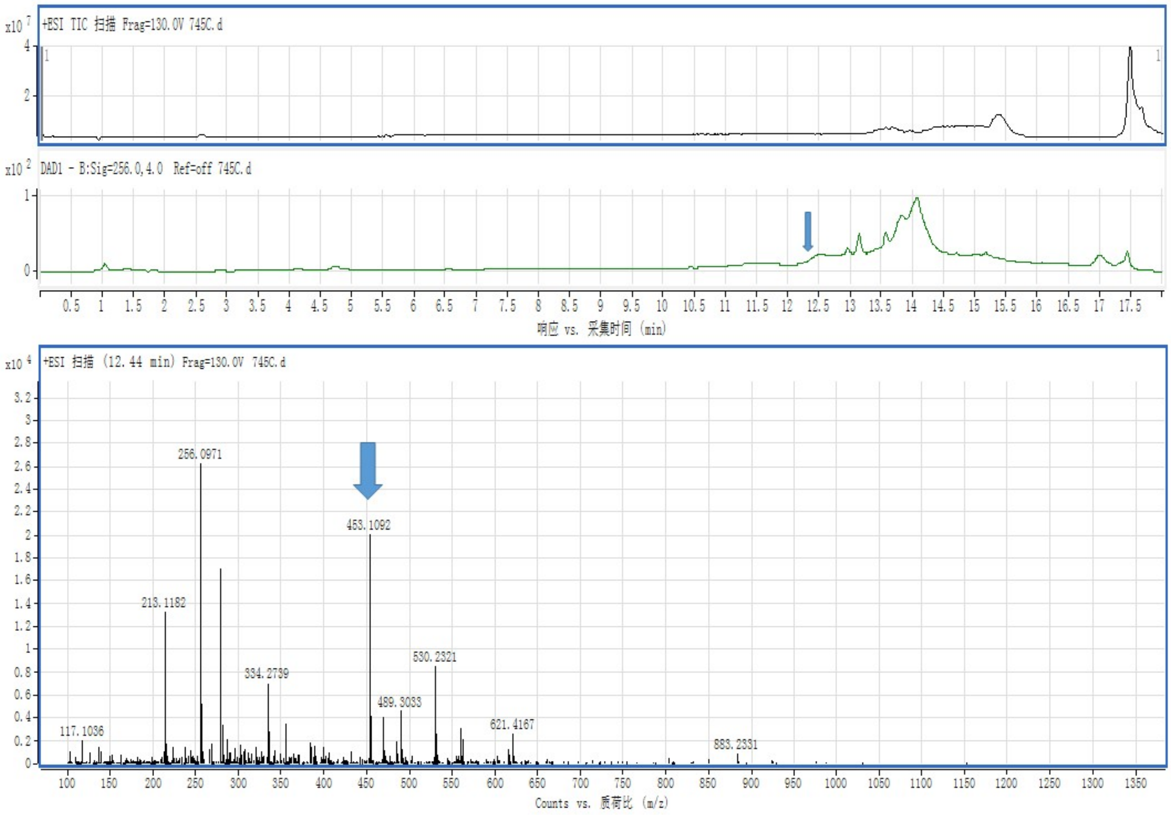

Supplement: Supplementary file 1 [file marinedrugs-16-00330-s001.pdf]
